# Microbial diversity in the arid and semi‐arid soils of Botswana

**DOI:** 10.1111/1758-2229.70044

**Published:** 2024-11-13

**Authors:** Coetzee Tidimalo, Ortiz Maximiliano, Jordaan Karen, Pedro H. Lebre, Olivier Bernard, Greve Michelle, Dikinya Oagile, Don A. Cowan

**Affiliations:** ^1^ Centre for Microbial Ecology and Genomics, Department of Biochemistry, Genetics and Microbiology University of Pretoria Pretoria South Africa; ^2^ Clemson University Genomics & Bioinformatics Facility Clemson University South Carolina USA; ^3^ Department of Plant and Soil Sciences University of Pretoria Pretoria South Africa; ^4^ Department of Environmental Science University of Botswana Gaborone Botswana

## Abstract

To date, little research has been conducted on the landscape‐scale distribution of soil microbial communities and the factors driving their community structures in the drylands of Africa. We investigated the influence of landscape‐scale variables on microbial community structure and diversity across different ecological zones in Botswana. We used amplicon sequencing of bacterial 16S rRNA gene and fungal internal transcribed spacers (ITS) and a suite of environmental parameters to determine drivers of microbial community structure. Bacterial communities were dominated by Actinomycetota (21.1%), Pseudomonadota (15.9%), and Acidobacteriota (10.9%). The dominant fungal communities were Ascomycota (57.3%) and Basidiomycota (7.5%). Soil pH, mean annual precipitation, total organic carbon, and soil ions (calcium and magnesium) were the major predictors of microbial community diversity and structure. The co‐occurrence patterns of bacterial and fungal communities were influenced by soil pH, with network‐specific fungi–bacteria interactions observed. Potential keystone taxa were identified for communities in the different networks. Most of these interactions were between microbial families potentially involved in carbon cycling, suggesting functional redundancy in these soils. Our findings highlight the significance of soil pH in determining the landscape‐scale structure of microbial communities in Botswana's dryland soils.

## Graphical abstract

Soil microbial community structures differ by ecological zones, with the Sandveld having significantly lower alpha diversity than the Wet‐Sandveld and Hardveld. The most important drivers of microbial composition and diversity are net plant productivity (NPP) and soil pH. Furthermore, soil pH influences bacteria–fungi interactions in these soils.

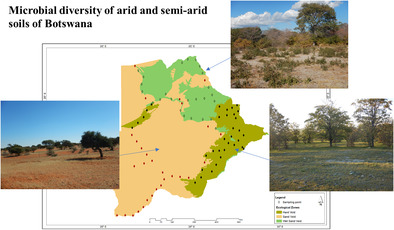

## INTRODUCTION

Drylands, which cover 41% of the world's land surface, include hyper‐arid, arid, semi‐arid and dry sub‐humid areas (Feng & Fu, 2013). According to model simulations, aridity is increasing significantly globally due to climate change and is leading to the expansion of global drylands (Feng & Fu, 2013; Huang et al., 2016). In addition, climate change is predicted to accelerate transitions from semi‐arid to arid ecosystems, inevitably impacting the interconnected structural and functional attributes of terrestrial ecosystems (Maestre et al., 2016). These ecosystem transformations may result in the loss of vegetation cover, a sharp decline in soil nutrients (carbon (C) and nitrogen (N) and a vegetation shift from grasslands and savannahs to shrublands (Berdugo et al., 2020; Delgado‐Baquerizo et al., 2013). Prolonged exposure to the adverse conditions in these ecosystems reduces microbial cellular and metabolic activity (Leung et al., 2020), invariably causing potentially detrimental changes to the soil microbiome (Maestre et al., 2015; Neilson et al., 2017). Soil microorganisms play important roles in ecosystem functions by participating in biogeochemical cycling (Fierer, 2017), improving plant health and productivity (Nadeem et al., 2014) and maintaining soil structure (Leifheit et al., 2014). As a result, alterations in these ecosystems may reduce the capacity of drylands to provide key ecosystem services.

Prevailing environmental conditions, including biotic and abiotic factors, determine microbial community structural and functional diversity (Bardgett & Van Der Putten, 2014; Fierer, 2017). A considerable body of research has highlighted the importance of edaphic, floristic and climatic variables in influencing soil microbial community composition and structure (Andrew et al., 2012; Bahram et al., 2018; Cowan et al., 2022; Egidi et al., 2019b; Lauber et al., 2008). The findings of these studies indicate that drivers of microbial community structure may differ with the spatial scale of the experiment and habitats (Bardgett & Van Der Putten, 2014). Indeed, one limitation of fine‐scale studies is the failure to incorporate cross‐biome effects (Delgado‐Baquerizo & Eldridge, 2019). Furthermore, the influence of some environmental variables is more evident at larger spatial scales (Bardgett & Van Der Putten, 2014).

Several recent studies demonstrate the importance of environmental factors and dispersal limitation in determining the structure and diversity of microbial communities in drylands at a landscape scale (Maestre et al., 2015; McHugh et al., 2017; Wang et al., 2017). Drylands are characterized by low and generally unpredictable precipitation levels, and high daily temperature fluctuations with intense solar radiation (Huang et al., 2016; Makhalanyane et al., 2015). Therefore, it is unsurprising that climatic variables are widely acknowledged as drivers of soil microbial structure and function in drylands. Aridity, mean annual temperature (MAT) and mean annual precipitation (MAP) have been identified as important variables negatively influencing microbial diversity, taxa distribution and relative abundance in dryland soils (Maestre et al., 2015; McHugh et al., 2017; Vásquez‐Dean et al., 2020). Moisture deficiencies in drylands directly influence microbial activities such as growth and respiration (Meisner et al., 2015), resulting in reduced physiological functioning and metabolic rates (Schimel, 2018). These factors indirectly influence microbial diversity by impacting soil pH, organic carbon content, and bulk density (Maestre et al., 2015; Yang et al., 2021). A growing body of research also emphasizes the importance of edaphic factors such as pH, organic carbon and total nitrogen in causing shifts in microbial community structure and diversity (Maestre et al., 2015; McHugh et al., 2017; Zeng et al., 2019).

Strong relationships are also observed between the microbial community structure and geographic distance (Wang et al., 2015, 2017; Zeng et al., 2019). Distance‐decay relationships indicate that the community composition of sites becomes less similar as geographic distance increases (Wang et al., 2015, 2017). These studies show that environmental filtering and dispersal limitations congruently influence soil microbial diversity. However, the relative importance of these two processes on microbial β‐diversity patterns varies depending on geographic scale, habitat, and taxa type (Chen et al., 2017; Wang et al., 2015, 2017). Furthermore, the importance of geographic distance on prokaryotic communities is indirect through the influence of soil properties and climate (McHugh et al., 2017).

While extensive research on soil microbial diversity has been carried out in many drylands of the world, drylands regions in Africa remain relatively unexplored (Vásquez‐Dean et al., 2020: Cowan et al., 2022). In Southern Africa, dryland expansion projections indicate an increase in the arid climate over most of Namibia and Botswana (Huang et al., 2017). Therefore, an improved understanding of biogeography and the drivers of microbial community structure in these areas will facilitate predictions of the possible alterations in ecosystem functioning due to desertification and may assist in the design of effective management strategies. To investigate microbial diversity and the effect of landscape variables on microbial community structure and diversity, we carried out a field survey across different ecological zones in Botswana. Botswana is classified as semi‐arid to arid (Batisani & Yarnal, 2010; Nkemelang et al., 2018) and is subject to high inter‐annual climate variability and periodic drought (Chipanshi et al., 2003). There are two major ecological zones in the country; the Sandveld and the Hardveld. The Sandveld covers 80% of the landmass (Winterbach et al., 2014) and is dominated by the arid Kalahari Desert in the country's southwestern region. The northern part of the Sandveld is further identified as the Wet‐sandveld (Atlhopheng et al., 2022; Sianga & Fynn, 2021). The mean annual precipitation of this ecological zone ranges from less than 200 mm in southwestern Botswana to 500 mm in the north (Thomas & Shaw, 1991). The Sandveld vegetation cover is predominantly semi‐arid savanna shrub‐woodland. The wetter northern parts are characterised by *Colophosphermum mopane*, whereas the southern area is predominated by various acacia species (Dougill et al., 2016; Ringrose et al., 2003). Land use in the Sandveld and Wet‐sandveld is predominantly communal and commercial livestock grazing, designated wildlife conservation areas and some small‐scale farming (Dougill et al., 2016; Winterbach et al., 2014). The semi‐arid Hardveld is mainly found in the eastern part of the country (Mphale et al., 2018). This ecological zone features rocky hill ranges, nutrient‐rich loamy, savannah grasslands, woodlands and patches of forest (Ringrose et al., 2002; Winterbach et al., 2014). Rainfall ranges from 350 to 650 mm per annum. It is therefore more suitable for agricultural activities and tends to have a high population density. Most of the land is used for crop production and livestock grazing (Ringrose et al., 2002). Previous studies on soil microbiology carried out in the country were conducted on a local scale (Elliott et al., 2014; Mhete et al., 2020), assessed a single taxonomic group (Law et al., 2007), or only detailed the physiological capabilities of soil microorganisms (Pule‐Meulenberg & Dakota, 2009).

We hypothesized that microbial communities found in the different Botswana ecological zones will be different and variations in microbial diversity will primarily be driven by climatic variables. To test this hypothesis, we used amplicon sequencing of bacterial 16S rRNA gene and fungal internal transcribed spacer (ITS) region to characterise microbial communities and assess the role of environmental variables as drivers of microbial diversity across the country. In doing so, we aimed to answer the following questions: (i) What microbial taxa dominate Botswana's soils on a landscape scale? (ii) How does soil microbial diversity and composition vary across the different ecological zones? (iii) What climatic, floristic, and edaphic factors best predict soil microbial structure and diversity on a landscape scale?

## EXPERIMENTAL PROCEDURES

### Study sites and soil collections

**FIGURE 1:**
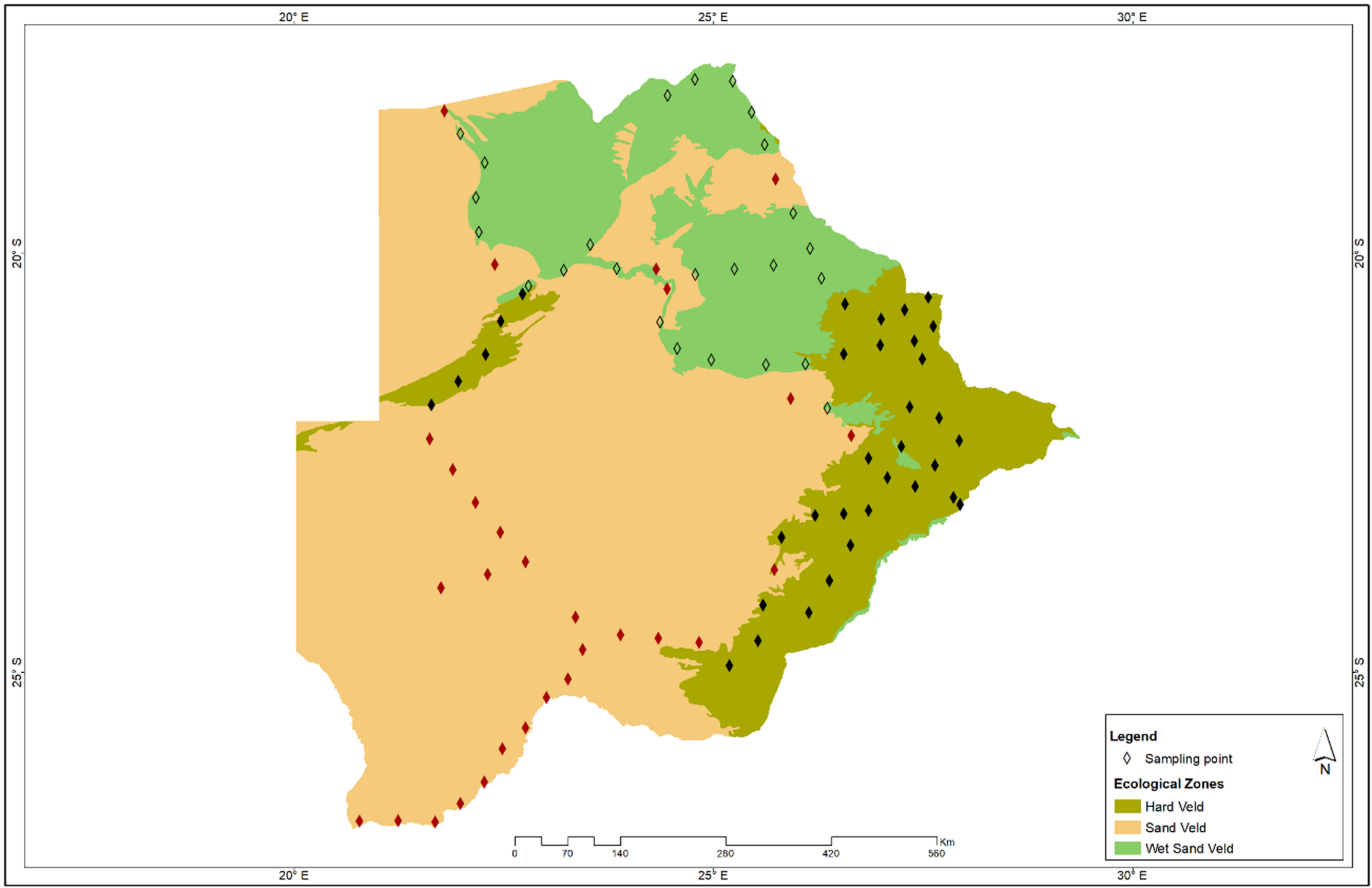
Map of Botswana showing the 89 sampling sites obtained from the Hardveld, Sandveld, and Wet‐sandveld ecological zones (Winterbach et al., 2014). Diamond symbols represent the sampling sites.

To provide coverage of the country's ecological and climatic variability, 89 sampling sites (34 from Hardveld, 30 from Sandveld, and 25 from wet‐sand veld) were selected at approximately 50 km intervals along transects totalling some 5200 km (Figure 1). Soil samples were collected in June 2018, and geographic coordinates for each sampling site were recorded. At each sampling location, a 100 m × 50 m plot was identified, and four ~200 g samples of 0–5 cm surface soil were collected in sterile Whirlpak® bags from each plot after the removal of surface plant litter. Samples were stored on ice during sampling and transportation to the Department of Environmental Science, University of Botswana, where they were stored at 4°C before being shipped to the Centre for Microbial Ecology and Genomics, University of Pretoria, for storage at −80°C. All samples were sieved (4 mm mesh) to remove plant roots and other debris. Each sample was subsequently divided into two parts: for soil physicochemical and microbial phylogenetic analyses.

### Climatic variables

Mean annual temperature (MAT) and annual precipitation (MAP) were obtained from the WorldClim2 Global Climate Database at a resolution of 30 arc‐seconds (Fick & Hijmans, 2017). The satellite‐derived 2‐band Enhanced Vegetation Index (EVI2) was used as a proxy for net plant productivity (NPP) in the sampled areas (Jiang et al., 2008; Pettorelli et al., 2005). Enhanced Vegetation Index (EVI2) data for June (2018) was obtained from the NASA EOSDIS Land Processes DAAC at a resolution of 500 m (Didan & Barreto, 2018). The aridity index (AI) was obtained from the CGIAR‐CSI GeoPortal (https://cgiarcsi.community) (Trabucco & Zomer, 2018). Information collation was based on the GPS location of each sampling site.

### Soil physicochemical analysis

Soil properties were determined according to the methods outlined in AgriLASA (2004). Fifteen soil physicochemical parameters were analysed for all samples (Table S1). Soil pH was measured using the slurry method at a 1:2.5 soil/water ratio. The pH of the supernatant was recorded with a calibrated benchtop pH meter (Crison Basic, +20, Crison, Barcelona, Spain). Soil total organic carbon (TOC) was determined according to the Black (1934) method. The Mehlich 3 test was used to determine the concentrations of the following extractable ions: sodium (Na), potassium (K), calcium (Ca), magnesium (Mg), manganese (Mn), phosphorus (P), iron (Fe), and aluminium (Al) (Mehlich, 1984). The extractable ion concentration was then quantified using ICP‐OES (inductively coupled plasma optical emission spectrometry, Spectro Genesis, SPECTRO Analytical Instruments GmbH & Co. KG, Germany). Soil particle size distribution (% sand, clay, and silt) was measured according to the Bouyoucos method. 70 g air‐dried and sieved soil samples were treated with 40 mL of hydrogen peroxide (30%) on a hot plate to remove organic matter. Hydrogen peroxide was added slowly until no effervescence occurred. Then 40 g air‐dried samples were mixed with 100 mL of 25% sodium hexametaphosphate (Colgan solution) and allowed to soak for 16 h. The suspension was mixed for 2 min, transferred to a 1 L sedimentary cylinder, and topped up to the 1 L mark with deionised water. After 2 h the first reading (R sand) was measured using a hydrometer that had been immersed in the suspension for 30 s. The second reading (R clay) was taken 6 h later. The silt fraction was calculated as the difference between the two measurements (Bouyoucos, 1962). Total nitrogen (TN) was determined using the catalysed high temperature combustion method (Dumas method) (Bremner, 1996).

### DNA extraction

DNA was extracted using the DNeasy PowerSoil Kit (QIAGEN, USA) following the manufacturer's instructions with minor modifications. The following modifications were made to the protocol: DNA was eluted by first adding 30 μL of preheated (55°C) solution C6 to the spin column, followed by 40 μL elution buffer to give a final volume of 70 μL DNA.

### Amplicon sequencing

Amplicon sequencing was performed by MRDNA laboratories (www.mrdnalab.com, Shallowater, TX, USA) according to in‐house protocols. Briefly, primers 515F (5′‐GTGYCAGCMGCCGCGGTAA‐3′) and 909R (5′‐CCCCGYCAATTCMTTTRAGT‐3′) were used to amplify the hypervariable V4/V5 region of the 16S rRNA gene in bacteria (Wang & Qian, 2009). The fungal internal transcribed spacers (ITS) region was amplified using ITS1F (5′‐CTTGGTCATTTAGAGGAAGTAA‐3′) and ITS4 (5′‐TCCTCCGCTTATTGATATGC‐3′) primers of the ITS‐1 and ITS‐2 regions respectively (Martin & Rygiewicz, 2005; White et al., 1990). A 30‐cycle polymerase chain reaction (PCR) reaction was performed using the HotStarTaq Plus Master Mix Kit (Qiagen, USA) for both 16S rRNA and ITS samples under the following conditions: 94°C for 3 min, followed by 30 cycles of 94°C for 30 s, 53°C for 40 s, and 72°C for 1 min, followed by a final elongation step at 72°C for 5 min. PCR products were electrophoresed on 2% (w/v) agarose gels to determine the presence and intensity of the amplification product. Amplicon products for each sample were pooled and purified using calibrated Ampure XP beads (Beckman Coulter Life Sciences, USA). Paired‐end sequencing (2× 300 bp) was performed on an Illumina MiSeq instrument following the manufacturer's guidelines.

### Bioinformatics analyses

Initial sequence processing and diversity analyses were conducted using QIIME2 (2017:6:0) (Bolyen et al., 2019). Raw sequences were demultiplexed, quality filtered, denoised and chimeric sequences removed using the DADA2 pipeline (Callahan et al., 2016). Low‐quality reads were removed by truncating reads to 290 bps for bacterial species and 190 bps for fungal species at an average quality score of 25. Quality sequences were then clustered into amplicon sequence variants (ASVs). Representative sequences were aligned using a multiple sequence alignment program (MAFFT) and a phylogenetic tree was generated with fasttree (Katoh & Toh, 2008). The taxonomic identity of the ASVs was determined using a trained SILVA 138 (release 12‐2019) database for prokaryotic species (Quast et al., 2012) and the UNITE fungal database (V7.2, release 11‐2018) (Kõljalg et al., 2005). SILVA 138 database was trained using q2‐feature‐classifier plugin in QIIME2 (QIIME2, 2020).

### Statistical analyses

Statistical analyses were performed using R v4.2.0 (R Core Team, 2013) using the following packages: phyloseq (McMurdie & Holmes, 2013), tidyverse (Wickham et al., 2019), caret (Kuhn, 2008), leaps (Lumley, 2013), car package (Dormann et al., 2013), and vegan (Oksanen, 2010).

A Kruskal–Wallis test was applied to determine the significant differences between the means of environmental factors between the ecological zones (McKight & Najab, 2010). Pairwise differences of different variables by ecological zones were assessed using Dunn's test (Dinno & Dinno, 2017). Spearman's correlations and corresponding *p* values were calculated for environmental variables. The *p*‐values were corrected for multiple testing using the Benjamini–Hochberg method (Benjamini & Hochberg, 1995).

Statistical analyses on community data were performed using rarefied (56,933 and 52,529 sequences per sample for 16S rRNA and ITS respectively), log_10_(*x* + 1) normalized data unless otherwise indicated. Phylum relative abundance and alpha diversity indices (richness and inverse Simpson) were determined with the phyloseq package. Although ASVs were used in this study to calculate species richness and diversity, we acknowledge that the use of ASV analyses can result in an overestimation of both species richness and diversity (Kauserud, 2023; Tedersoo et al., 2022). The Kruskal–Wallis test was used to determine significant differences in phylum relative abundances and alpha diversity metrics between the different ecological zones. Environmental predictors of microbial alpha diversity were identified using best subset regression (McLeod & Xu, 2010) in the tidyverse, caret, and leaps packages. The data was first tested for multicollinearity with the *vif* function in the car package. The best models were selected based on: (i) high adjusted *R* ^2^ values, (ii) Mallow Cp value that is close to the number of predictor variables, and (iii) goodness of fit of the residual plot. The relationship between environmental parameters and alpha diversity indices was further visualised using generalized linear regressions (Fox, 2015). The significance of the models was tested using ANOVA (Underwood, 1997).

Beta diversity was assessed using Principal Coordinates Analysis (PCoA) (Ramette, 2007) based on Bray–Curtis distance matrices, with the *ordinate* function in the vegan package. Permutational Multivariate Analyses of Variance (PERMANOVA), with 9999 permutations, was used to test for significant differences between ecological zones and pH groups using the *adonis2* function (Anderson, 2001). Variations within communities were determined by distance‐based tests for homogeneity using the *betadisper* function (Anderson, 2006).

Environmental drivers of microbial community structure were identified using redundancy analysis (RDA) in the vegan package (Legendre & Anderson, 1999). Community and environmental data were Hellinger transformed and *z*‐score standardized, respectively. The best models were selected using both the forward and backward procedures. Predictor variables with a variance inflation factor (VIF) > 10 were excluded from the final model. ANOVA was subsequently used to test for significance (Monte Carlo permutation test, 999 permutations) of the final model and individual predictor variables. Spearman's correlation analysis was used to determine the relationships between the relative abundance of major phyla with all the environmental variables. *p*‐Values were corrected for multiple testing using the Benjamini–Hochberg method (Benjamini & Hochberg, 1995).

Variation partitioning analysis (VPA) was used to assess the influence of edaphic, climatic, and spatial factors on community variation (Legendre, 2008; Peres‐Neto et al., 2006). Spatial variables were generated from the longitude‐latitude coordinates of each sampling point using principal coordinates of neighbour matrices (PCNM) (Borcard & Legendre, 2002) with function *pcnm*. The best set of variables explaining variation in community composition was determined by distance‐based redundancy analysis (db‐RDA), based on Bray–Curtis dissimilarity. The models were identified with the *capscale* function based on Bray–Curtis dissimilarity, followed by the selection of predictor variables using the *ordistep* function with forward selection. The *varpart* function was then used to examine community variation partitioning. The significance of the models was subsequently tested using ANOVA (Monte Carlo permutation test, 999 permutations). A partial residual plot was used to assess the effect of distance on Bray–Curtis.

Co‐occurrence networks were constructed between bacterial and fungal communities at different pH categories (acidic [pH 4.5–6.5, *n* = 30], alkaline [pH 7.5–10.3, *n* = 30], and neutral [pH 6.6–7.3, *n* = 29]) to determine potential relationships among taxa (U.S Department of Agriculture, 2022). The bacterial and fungal ASVs were first grouped according to pH categories. Each dataset was filtered by retaining all ASVs that occurred more than five times in at least 10% of samples (i.e., the core microbiome) using the phyloseq package. After this filtering step, we obtained a total of 1018 ASVs (822 bacterial and 191 fungal) in the acidic, 1006 ASVs (773 bacterial and 233 fungal) in the alkaline and 895 ASVs (636 bacterial and 259 fungal) in the neutral soils. Spearman correlations were determined for the filtered datasets using the absolute abundance of ASVs and *p*‐values were adjusted with the Benjamini–Hochberg procedure to minimize the possibility of false‐positive results (Benjamini & Hochberg, 1995). Spearman correlations with coefficient values (*r*) >0.7 or < −0.7 and *p* < 0.01 were considered significant and selected for bacteria–fungi co‐occurrence analysis (Jordaan et al., 2019). These were then translated into networks in Cytoscape 3.9.1 (Shannon et al., 2003). Network topology properties were calculated using the NetworkAnalyzer tool (Doncheva et al., 2012). Modular structures and clusters of highly interconnected nodes in each network were identified with the MCODE application tool in Cytoscape (Bader & Hogue, 2003). Keystone taxa were classified as having the highest degree (≥15) and cluster coefficient (>0.20) (Jordaan et al., 2019). The networks were further visualised using the interactive platform Gephi (v.0.9.7) (Bastian et al., 2009).

## RESULTS

### Physico‐chemical analysis

The soil physicochemical and climatic variables collected in this study are presented in Table 1. Most environmental variables significantly (*p* < 0.05) differed across the Hardveld, Sandveld and Wet‐sandveld. Soils from the Sandveld zone had statistically significant (*p* < 0.05) lower nutrient concentrations (TN, TOC, K, Ca, and Mg) and MAP than the other zones. Significantly higher (*p* < 0.05) NPP, MAT and pH content were observed in the Wet_sandveld soils than in other ecological zones. Hardveld soils had higher P, Al, and Fe concentrations than the Sandveld and the Wet‐sandveld zones. All soils were oligotrophic, as indicated by low soil TOC (0.06%–3.03%) and TN (0.01%–0.44%) levels. Soil pH across the sampling sites significantly (*p* < 0.05) correlated with MAP, MAT, soil K, Mg, Mg, Na, Mn, Fe, and Al content (Table S2). TOC and TN were significantly (*p* < 0.05) positively correlated with all the variables except MAT.

**TABLE 1:** Mean values for the environmental variables for the soils across the sampling sites.

	Hardveld	Sandveld	Wet‐sandveld	*p*‐Value
pH	6.65 ± 0.78a	7.14 ± 1.18a	7.83 ± 1.01b	3.38 × 10^−4^***
Carbon–nitrogen ratio	12 ± 3.06a	12.9 ± 4.25a	14.8 ± 5.33a	3.20 × 10^−1^
Concentration (mg/kg) of:				
Potassium (K)	193 ± 192a	100.0 ± 298.0b	168.0 ± 210.0a	6.14 × 10^−6^***
Calcium (Ca)	1497 ± 1389a	903.0 ± 1263.0b	3225.0 ± 2835.0a	1.00 × 10^−4^***
Magnesium (Mg)	280 ± 277a	89.6 ± 39.4b	349.0 ± 570.0a	9.98 × 10^−5^***
Sodium (Na)	27.8 ± 23.9a	528 ± 2809b	193 ± 759a	4.69 × 10^−5^***
Phosphorus (P)	15.7 ± 13.1a	10.2 ± 8.3b	8.3 ± 5.8b	5.76 × 10^−4^***
Manganese (Mn)	66.8 ± 40a	18.1 ± 20.8b	38.8 ± 29.9c	1.19 × 10^−8^***
Aluminium (Al)	375 ± 148a	164 ± 64.5b	201.0 ± 123.0b	3.09 × 10^−9^***
Iron (Fe)	79.2 ± 96.6a	29.3 ± 7.65b	33 ± 17.9b	1.69 × 10^−8^***
% of:				
Total organic carbon (TOC)	0.87 ± 0.48a	0.31 ± 0.13b	0.76 ± 0.53a	1.02 × 10^−10^***
Total nitrogen (TN)	0.09 ± 0.08a	0.03 ± 0.01b	0.06 ± 0.05a	4.67 × 10^−9^***
Sand	76.7 ± 14.9a	92.3 ± 4.62b	80.1 ± 20.4a	2.98 × 10^−8^***
Silt	3.56 ± 9.0a	0.33 ± 0.18b	2.04 ± 3.4a	3.0 × 10^−3^***
Clay	19.8 ± 9.24a	7.7 ± 4.6b	17.8 ± 181a	2.07 × 10^−8^***
NPP	2801 ± 381a	2534 ± 758a	3228 ± 3133b	1.05 × 10^−4^***
MAT	199 ± 32.4a	203 ± 8.89a	208 ± 39.4b	8.53 × 10^−6^***
MAP	433 ± 37.3a	358 ± 83.4b	437 ± 72.1a	4.12 × 10^−6^***
Aridity index	0.18 ± 0.03a	0.14 ± 0.03b	0.17 ± 0.04a	8.64 × 10^−7^***

Values are means (±standard deviation). Significant differences are based on the Wilcoxon rank‐sum test and are represented by asterisks as follows: ***p < 0.001, **p < 0.01, *p < 0.05. Pairwise differences were generated using Dunn's test.

### Microbial community composition

**FIGURE 2:**
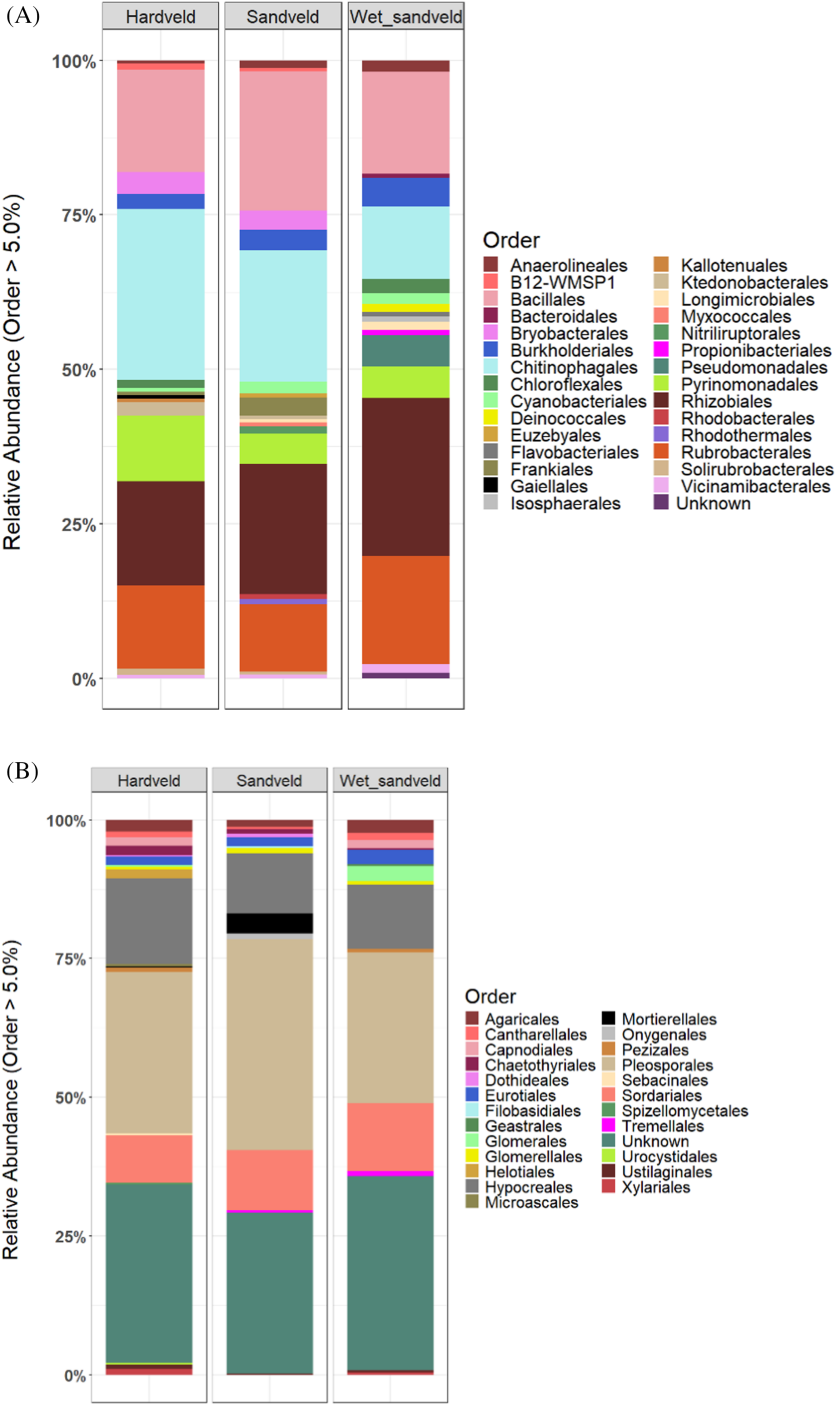
Relative abundance of the major (A) bacterial and (B) fungal phyla (≥5%) in Hardveld, Sandveld and Wet‐sandveld ecological zones.

Sequencing results yielded a total of 7,077,626 bacterial and 8,024,482 fungal amplicon sequences following data quality filtering, denoising and chimera removal. Sequence clustering into ASVs and subsequent rarefaction resulted in 37,643 bacterial and 13,815 fungal ASVs. Bacterial ASVs were assigned to 44 phyla (one unassigned) while fungal ASVs were assigned to a total of 16 phyla (two unassigned). Approximately 18.9% of the fungal reads could not be assigned to any known phylum. The most abundant bacterial phyla identified across all the samples were Actinomycetota (21.1%), Pseudomonadota (15.9%), Acidobacteriota (10.9%), Chloroflexota (8.6%), Bacteroidota (7.6%), Bacillota (7.1%), Planctomycetota (3.2%), Myxococcota (2.4%), Gemmatimonadota (2.1%), and Verrucomicrobiota (1.0%) Figure S1A). Similar phylum‐level community composition was observed when comparing the Hardveld, Sandveld and Wet‐sandveld communities separately (Figure S1A). However, significantly higher relative abundances of Planctomycetota (Kruskal–Wallis chi‐squared = 10.904, df = 2, *p*‐value = 0.004) and Gemmatimonadota (Kruskal–Wallis chi‐squared = 9.753, df = 2, *p*‐value = 0.008) were observed in the Wet‐sandveld samples. The Hardveld samples were characterised by a significantly higher relative abundance of Bacteroidota (Kruskal–Wallis chi‐squared = 11.307, df = 2, *p*‐value = 0.00351‐). At the order level, Chitinophagales (6.36%), Bacillales (6.28%), and Rhizobiales (6.12%) dominated the communities across the different ecological zones (Figure 2A). The relative abundance of Chitinophagales (Kruskal–Wallis chi‐squared = 22.669, df = 2, *p*‐value = 1.20 × 10^−5^) significantly differed between samples in the different ecological zones. A significant increase in the relative abundance of Frankiales (Kruskal–Wallis chi‐squared = 12.188, df = 2, *p*‐value = 0.002‐) was observed in the Sandveld samples. The relative abundance of Vicinamibacterales (Kruskal–Wallis chi‐squared = 10.78, df = 2, *p*‐value = 0.005) was significantly higher, while that of Bryobacterales (Kruskal–Wallis chi‐squared = 22.055, df = 2, *p*‐value = 1.62 × 10^−5^) was significantly lower in the Wet‐sandveld samples than other ecological zones.

Ascomycota, accounted for 57.3% of all ITS amplicon sequences, followed by Basidiomycota (7.5%), Glomeromycota (1.6%), and Mortierellomycota (1.3%). Ascomycota and Basidiomycota dominated the communities across the three ecological zones (Figure S1A). Conversely, an increase in the relative abundance of Mortierellomycota was observed in the Sandveld (Kruskal–Wallis chi‐squared = 11.701, df = 2, *p*‐value = 0.003), while Glomeromycota was more prominent in the Wet‐sandveld (Kruskal–Wallis chi‐squared = 18.711, df = 2, *p*‐value = 8.65 × 10^−5^). Major fungal orders detected across the different ecological zones were Pleosporales (22.65%), Hypocreales (9.48%), and Sordariales (8.20%) (Figure 2B). A significant decrease in the relative abundance of Mortierellales (Kruskal–Wallis chi‐squared = 11.239, df = 2, *p*‐value = 0.004) and Glomerales (Kruskal–Wallis chi‐squared = 20.759, df = 2, *p*‐value = 3.11 × 10^−5^) was observed in the Wet‐sandveld and Sandveld samples, respectively. The relative abundance of Mortierellales was 0.05%, 0.30% and 1.0% across the Wet‐sandveld, Hardveld, and Sandveld ecological zones, respectively. Glomarales relative abundance was 0.16%, 0.37%, and 0.74% in the Sandveld, Hardveld, and Wet‐sandveld zones respectively.

### Soil microbial diversity

**FIGURE 3:**
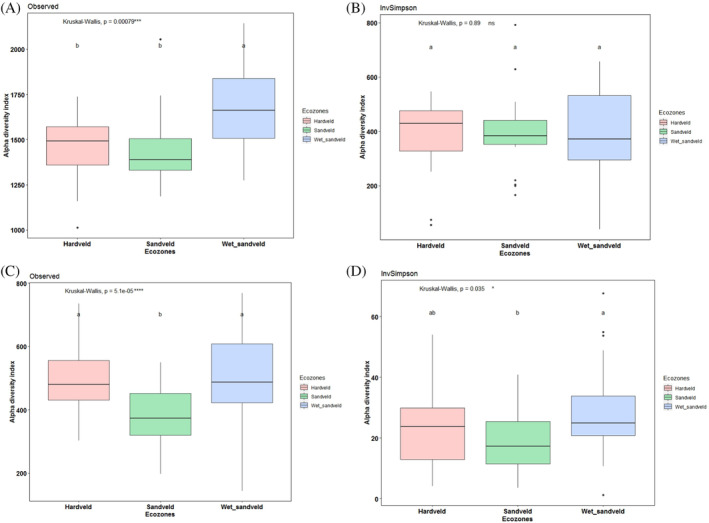
Alpha diversity indices (Observed and InvSimpson) for (A and B) bacterial and (C and D) fungal communities. Kruskal–Wallis test was performed for significant differences in the alpha diversity of the ecological zones. Significant levels are represented as follows: ***p < 0.001, ·*p* < 0.1.

Bacterial species richness ranged between 1012 and 2143 across the ecological zones. Sandveld had significantly lower bacterial richness (chi‐squared = 9.51, df = 2, *p*‐value = 0.009) than Hardveld and Wet‐sandveld (Figure 3A). However, species diversity showed no significant difference among the ecological zones (Figure 3B). Fungal species richness ranged from 135 to 750 across all samples. Overall, the Wet‐sandveld were richer (chi‐squared = 19.78, df = 2, *p*‐value = 5.07 × 10^−5^) and more diverse based on the inverse of the Simpson (InvSimpson) diversity index test results (chi‐squared = 6.72, df = 2, *p*‐value = 0.03) than the Sandveld (Figure 3C,D). The best subset regression identified Mg, Na, NPP, and soil pH as the best predictors of bacterial species richness across the ecological zones (Table S3). However, the influence of Mg was not statistically significant (*p* < 0.05) and was not used for downstream analysis. Bacterial species richness positively correlated with Na, NPP, and soil pH (Figure S2A–C). The best predictors of species richness for fungal communities were NPP, Mn, Na, and soil TOC, with Mn being non‐significant (*p* < 0.05) (Table S4). Fungal species richness increased with NPP, Na, and soil TOC (Figure S2D–F). Assessment of the influence of these variables per ecological zone indicates that NPP's influence on fungal species richness was only statistically significant (*R* ^2^ = 0.303, *p*‐value = 9.63 × 10^−4^) in the Sandveld samples. Conversely, Mn positively influenced fungal species richness in the Hardveld samples only (*R* ^2^ = 0.160, *p*‐value = 0.016).

### Drivers of soil community composition

**FIGURE 4:**
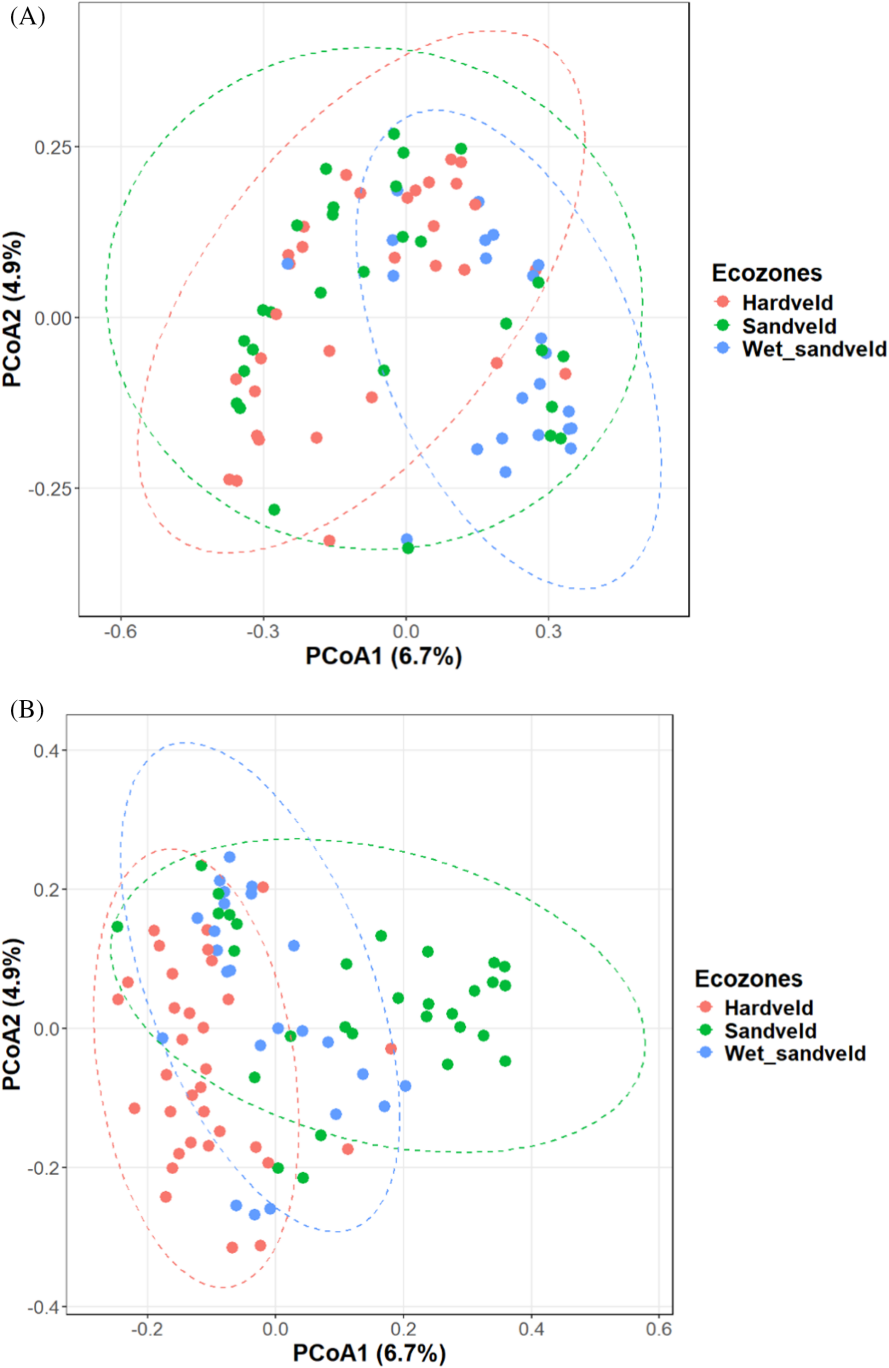
Principal coordinates analysis (PCoA) plots of (A) bacterial communities as related to and (B) fungal communities as related to ecological zones based on Bray–Curtis similarity matrices respectively.

**FIGURE 5:**
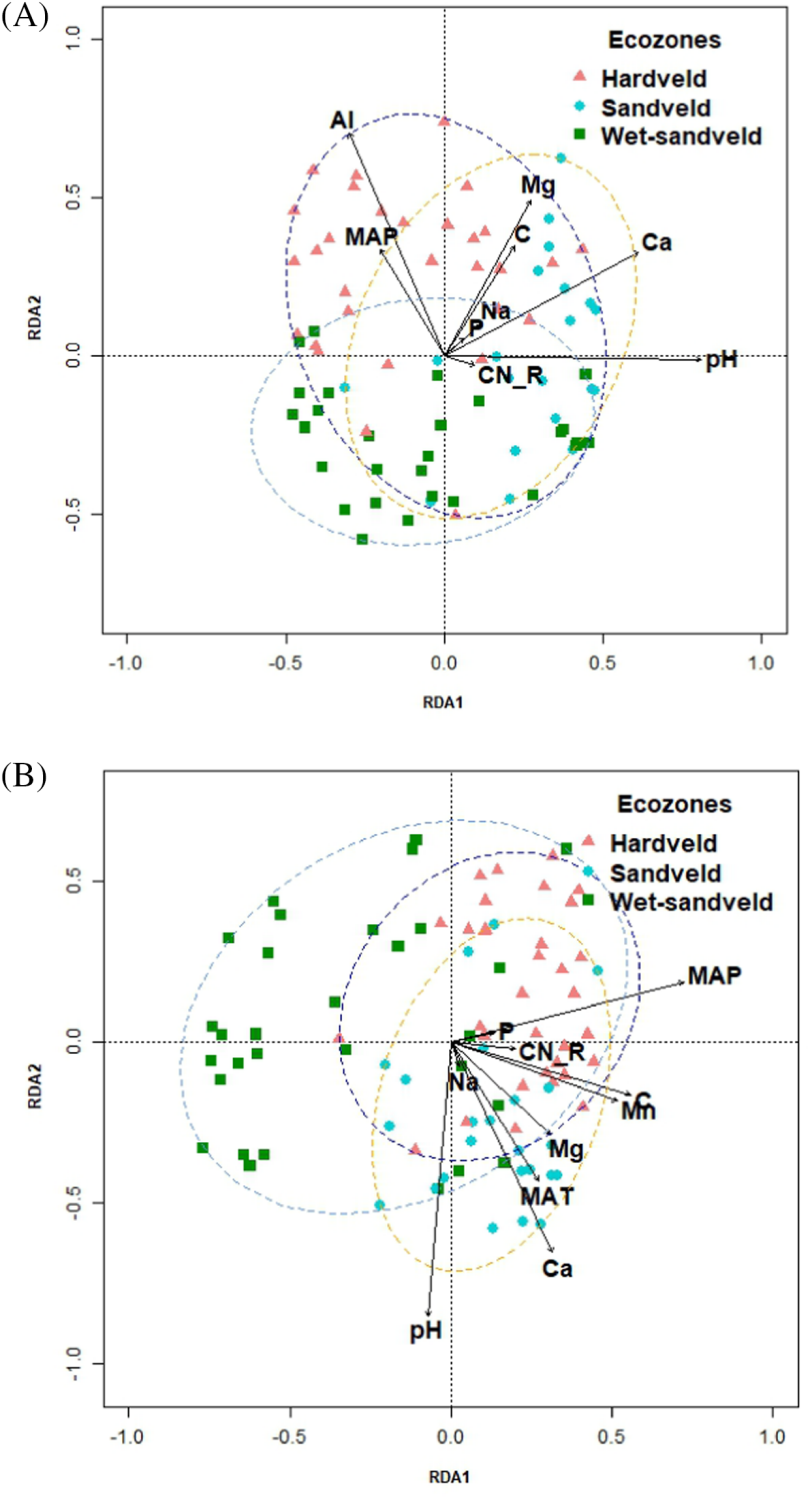
Redundancy analysis (RDA) plot showing relationships between (A) bacterial and (B) fungal community structures and environmental factors. The plot only shows edaphic and environmental variables that significantly influence community diversity.

The PCoA plot based on Bray–Curtis distance matrices did not exhibit clustering of bacterial communities according to ecological zones (Figure 5A). However, Permanova performed on the bacterial data indicated significantly different communities across different ecological zones (*R* ^2^ = 0.100, *p*‐value = 0.0001). This was supported by an insignificant betadisper (*p*‐value = 0.926) test result, suggesting that the differences observed between ecological zones are meaningful and not simply due to random variation within the groups. For fungal communities, PCOA plots showed distinct clustering according to soil ecological zones (Figure 4B), and this was confirmed by a significant Permanova (*R* ^2^ = 0.060, *p*‐value = 0.0001) test and an insignificant betadisper (*p*‐value = 0.060). These results suggest diverse fungal and bacterial communities per ecological zone.

### Impact of physicochemical parameters on microbial community structure

RDA was used to identify the environmental factors that influence the structure of soil microbial communities across Botswana. Drivers of community structure differed between bacterial and fungal communities. Edaphic factors significantly influencing bacterial community structure (*p* < 0.05) were soil pH, C:N ratio, Al, Ca, Mg, TOC, Na, and P content (Figure 5A and Table S5). The only significant climatic driver of bacterial community structure was MAP (*p*‐value = 0.006). The major edaphic drivers of fungal community structure (*p* < 0.05) were soil pH, C:N ratio (*p*‐value = 0.004) and soil Ca, Mn, Mg, C, Na, and P content (Figure 5B and Table S6). Conversely, the dominant climatic drivers of fungal diversity were MAP (*p*‐value = 0.001) and MAT (*p*‐value = 0.001). Soil pH was the most important common predictor of bacterial and fungal communities (Figure 5A,B). Overall, the combination of the environmental parameters explained 29.0% and 20.4% of the bacterial and fungal community variation, respectively (*p* < 0.05).

The relative abundances of the dominant (relative abundance ≥1%) bacterial and fungal phyla significantly correlated with most of the environmental variables (*p* < 0.05) (Figure S3). The abundance of Gemmatimonadota, Myxococcota, Planctomycetota, Armatimonadota, and Pseudomonadota positively correlated with soil pH, while the opposite pattern was observed for Verrucomicrobiota, Bacteroidota, and Bacillota (Figure S3A). Soil Al content correlated positively with the abundance of Verrucomicrobiota, Actinomycetota, Acidobacteriota and Chloroflexota and negatively with Pseudomonadota. For fungal phyla, the relative abundance of Mortierellomycota was negatively associated with MAT, soil Na, Ca content, and soil pH (Figure S3B), while that of Ascomycota was negatively associated with soil TOC. The relative abundance of Glomeromycota positively correlated with MAP, MAT, soil TOC, soil Mn, Mg, Ca, and Na and negatively correlated with soil P content. In contrast, the relative abundance of Basidiomycota was not significantly predicted by assessed environmental variables.

**FIGURE 6:**
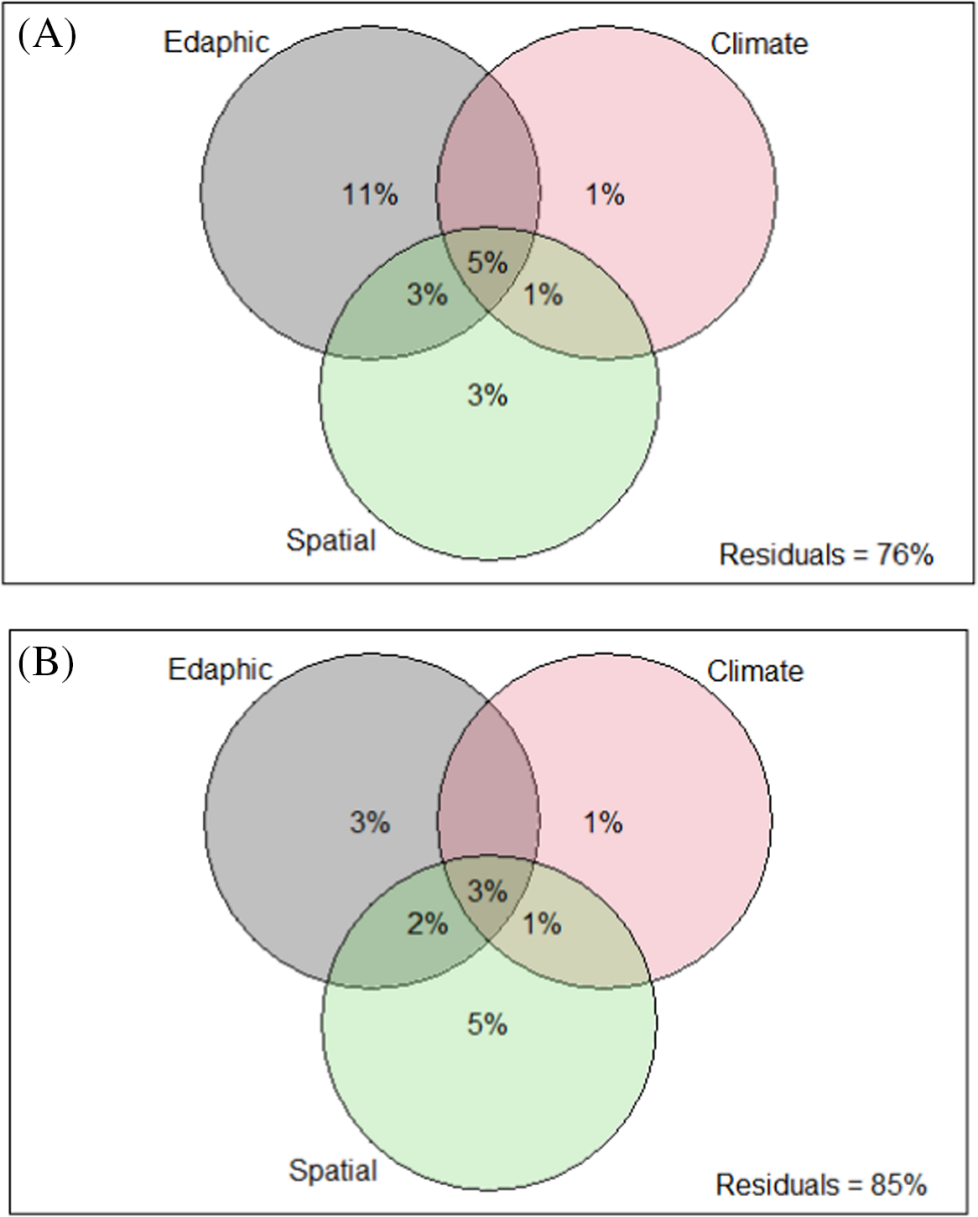
Variation partitioning analysis of (A) bacterial and (B) fungal community structure explained by edaphic, climatic, and spatial factors, and their interactions. The fraction of the variation is shown by adjusted *R* ^2^ values, *p*‐value = 0.001.

The percentages of community variation attributed to edaphic, climatic and spatial factors were determined using variation partitioning analysis (Figure 6). For bacterial communities, these three factors explained 24% of the variation in community composition, with 9.0% of this being shared among the three factors (Figure 6A). Variation was significantly (*p*‐value = 0.001) explained by edaphic parameters (11%), followed by spatial (3.0%) and climatic (1.0%), respectively. A large proportion (74%) of the variation remained unexplained. For fungal communities, variation was mainly explained by spatial variables (5.0%), followed by edaphic (3.0%) and climatic factors (1.0%) (Figure 6B). A total of 6.0% of community variation was shared among the three factors, whereas 85% of the variation remained unexplained. There was no interaction detected between edaphic and climatic factors of either bacterial or fungal communities. The Bray–Curtis abundance‐based dissimilarity index was used to calculate pairwise community dissimilarity amongst all sampling sites. The influence of geographic distance on community dissimilarity was statistically significant (*r*: 0.003, *p* < 0.001) for fungal communities only (Figure S4). While the influence of geographic distance on community dissimilarity was statistically significant, the low *r*‐value indicates that these findings lack practical significance or interpretability.

### Detection of microbial biomarkers

**FIGURE 7:**
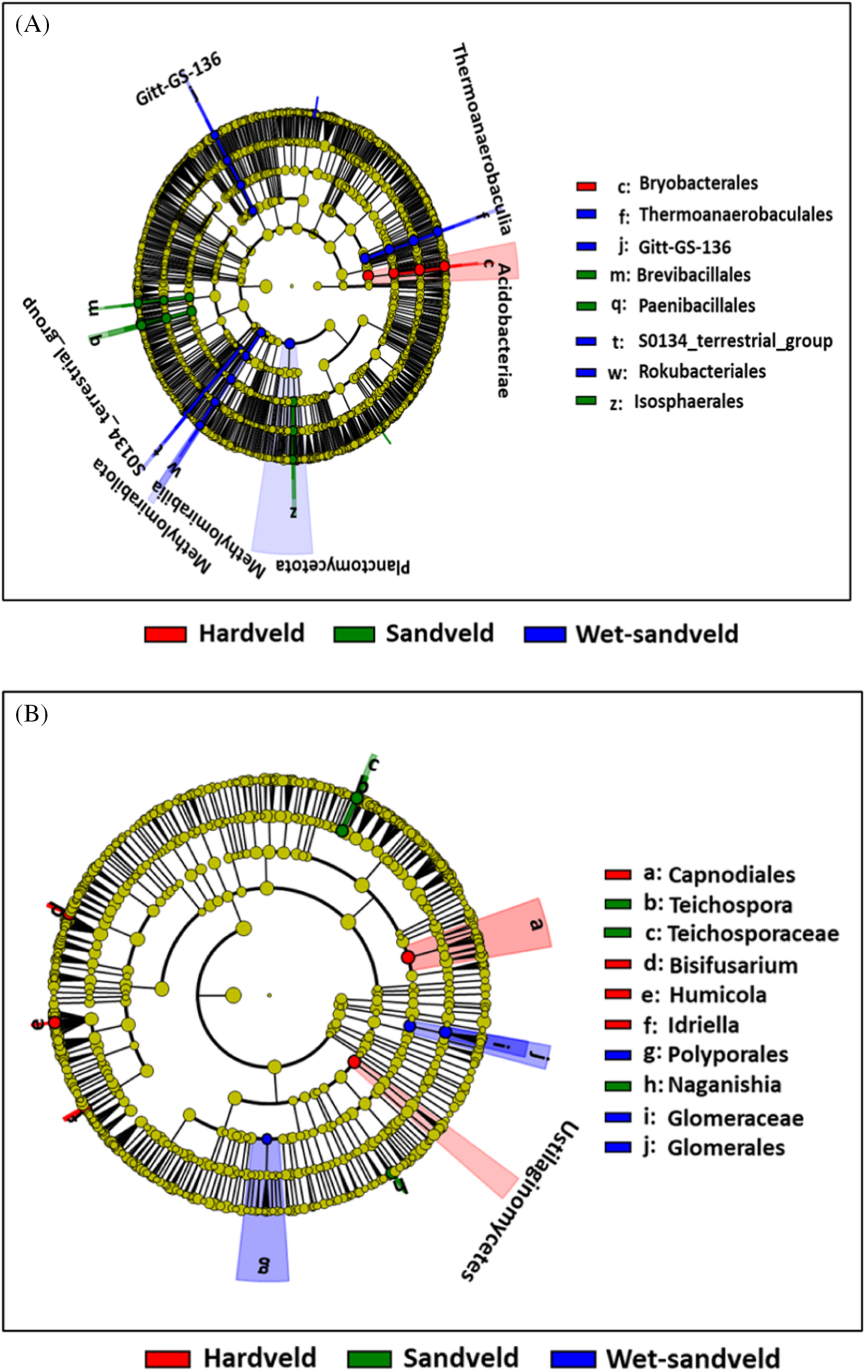
Taxonomic cladogram showing differentially abundant taxa for bacterial and fungal communities in different ecological zones. Phylogenetic levels are represented by the rings (from inner to outer ring): phylum, class, order, family and genus. (A) Bacterial communities. (B) Fungal communities.

Differentially abundant clades were determined as a means of identifying microbial biomarkers in the ecological zones. A total of 34 statistically significant differentially abundant bacterial taxa were identified across all soil samples (*p* < 0.05): 4 were from Hardveld, 11 from Sandveld and 19 from Wet‐sandveld samples. Only 2 of the 34 biomarkers, Plactomycetota and Methylomirabilota, were classified to phylum level (Figure 7A). At family level, biomarkers were Thermoanaerobaculaceae, SO134‐terrestrial‐group, Rokubacteriales and Gitt‐GS‐136 in the Wet‐sandveld; Paenibacillaceae, Isosphaeraceae, and Brevibacillaceae in Sandveld; and Bryobacteraceae in Hardveld soils. Of the 11 fungal taxa biomarkers identified, 5 were from Hardveld, 3 from Sandveld and 3 from Wet‐sandveld soils. There was only one class‐level biomarker: Ustilagomycetes (Figure 7B). Hardveld soils were associated with members of the orders Capnodiales and Hypocreales (*Bisifusarium*) while members of Pleosporales (*Teichospora*) and Filobasidiales (Naganishia) dominated the Sandveld. Glomeraceae were uniquely associated with Wet‐sandveld soils (Figure S5A).

### Co‐occurrence between bacterial and fungal domains

**FIGURE 8:**
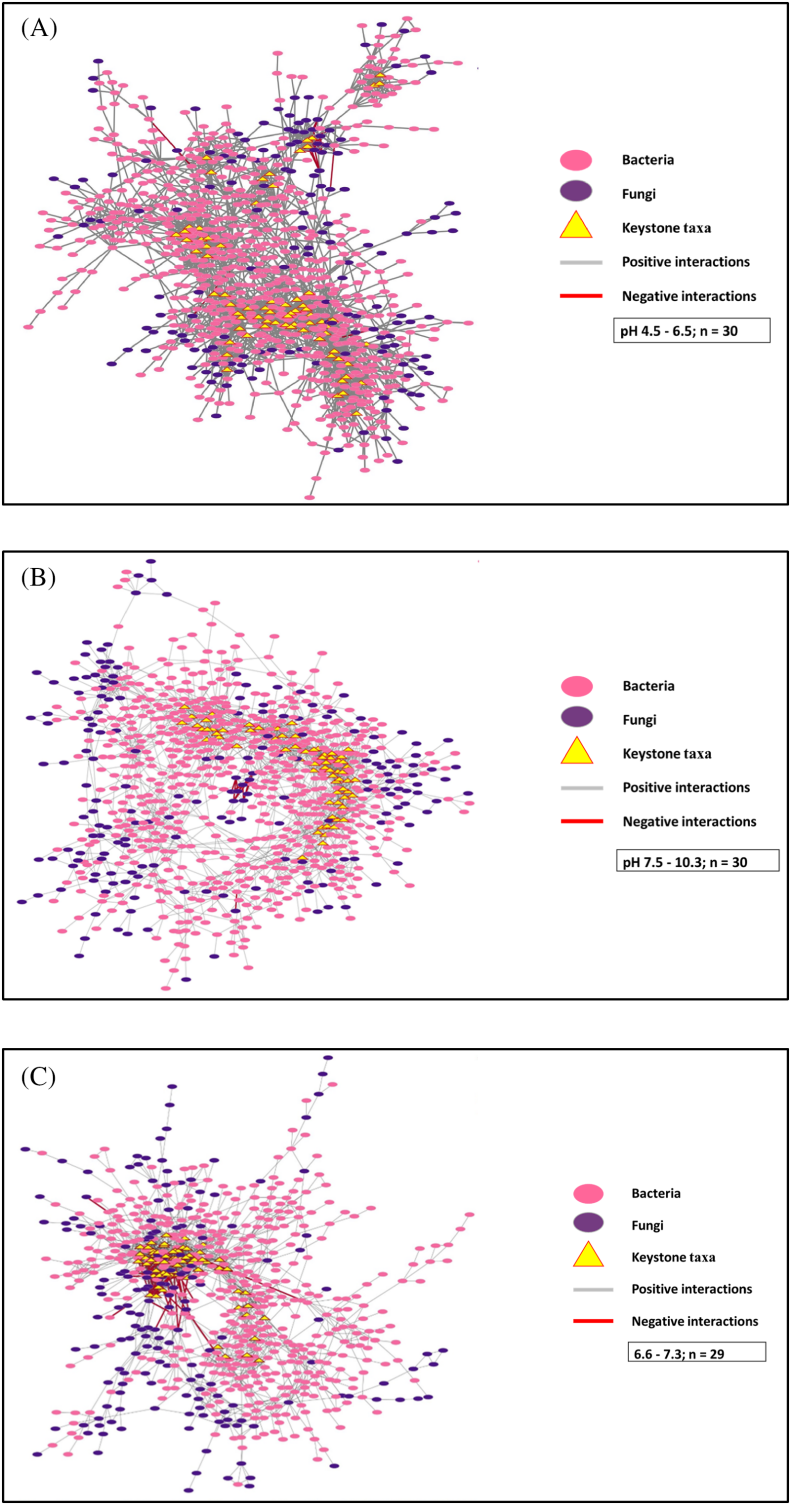
Bacteria–fungi co‐occurrence networks in (A) acidic, (B) alkaline, and (C) neutral soils. A grey edge represents a positive correlation, and a red edge represents a negative correlation.

Considering the observed importance of pH as a potential driver of soil microbial community composition across Botswanan soils, the influence of this variable on bacterial–fungi interactions was further investigated using a co‐occurrence network analysis. The co‐occurrence network topological properties of the networks are outlined in Table S7, which are similar for all three networks. For example, all the networks had a high modularity index (>0.4), suggesting they had a modular structure. Bacterial nodes dominated the co‐occurrence networks across all pH categories and most interactions were positive (99%, 99% and 97% in acidic, alkaline and neutral, soils respectively) (Figure 8A–C). Most of the negative interactions were fungi–fungi and these were mainly observed in neutral soils (3%). We identified 238, 316 and 371 bacteria–fungi links in acidic, alkaline and neutral networks, respectively. The nodes in the networks across the three pH categories were all assigned to 23 phyla, including 17 bacterial and 6 fungal phyla. The bacterial phyla primarily consisted of Actinomycetota (20.00%), Pseudomonadota (15.64%), Acidobacteriota (10.75%), and Bacteroidota (8.00%), while the major fungal phylum was Ascomycota (15.11%). The relative proportion of the nodes of these dominant microbial phyla (bacteria and fungi) did not vary across the three pH networks. At the family level, Chitinophagaceae, Beijerinckiaceae, and Rubrobacteriaceae had relatively high abundance across the different networks. Chaetomiaceae was the most common fungus found across all the networks. Bacteria–fungi interactions varied with soil pH, and most of these interactions were observed in the neutral pH network (Figure S6). These interactions were mainly derived from Ascomycota interacting with bacterial phyla. Ascomycota had a relatively high co‐occurrence with Actinomycetota, Acidobacteria, and Chloroflexota in the acidic network; co‐occurred with Actinomycetota, Chloroflexota, and Pseudomonadota in the alkaline network; and finally, with Chloroflexota, Actinomycetota, and Bacillota in the neutral network.

### Modules of networks

**FIGURE 9:**
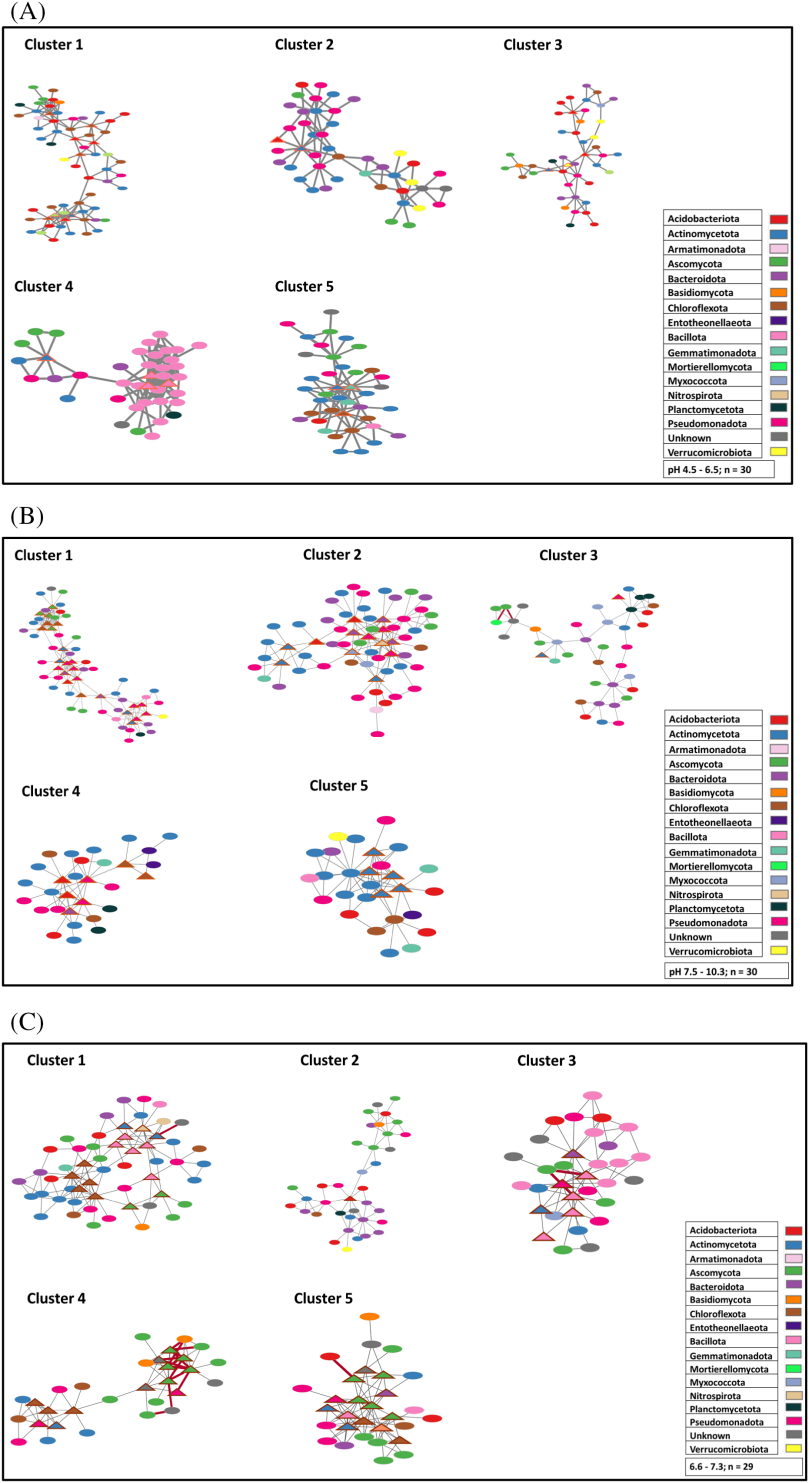
Co‐occurrence network clusters of bacterial and fungal communities in (A) acidic, (B) alkaline, and (C) neutral soils. Node colour represents different phyla.

MCODE analysis revealed several modules in the different networks. Our analysis focused on the top five clusters in each network, which were selected based on the high number of nodes and bacteria–fungi links. The composition of the modules varied within each network and between pH networks. Bacteria–bacteria interactions dominated all modules in the acidic network (Figure S7A) and alkaline network (Figure S7B). In the neutral network, modules I–III were dominated by bacteria–bacteria interactions, while modules IV–V had an equal occurrence of bacteria–bacteria and fungi–fungi interactions (Figure S7C). The major taxa in the acidic network were Acidobacteriota, Actinomycetota, and Chloroflexota, which co‐occurred with diverse bacterial and fungal phyla (Figure 9A). Module 4 was characterised by Bacillota–Bacillota interactions. In the alkaline network modules, Pseudomonadota, Actinomycetota, and Bacteroidota co‐occurred with different bacterial phyla and Ascomycota (Figure 9B). In the neutral network, the modules were characterised by interactions between Ascomycota, Bacillota, Actinomycetota, and Chloroflexota with diverse microbial phyla (Figure 9C). Our results indicate that although some fungal families, such as Chaetomiaceae, Nectriaceae, and Didymellaceae, occurred in modules across the different networks, they interacted with phylogenetically diverse bacterial families in each network. At the family level, distinct network‐specific fungi–bacteria interactions were observed in the different network modules. Nocardioidaceae, Bryobacteraceae, and Geodermatophilaceae interacted with Chaetomiaceae and Nectriaceae in acidic soils. In alkaline soils, Chitinophagaceae, Roseiflexaceae and AKIW781 interacted with Chaetomiaceae and Sporormiaceae. The neutral network was characterised by associations between Bacillaceae, Roseiflexaceae, and Chitinophagaceae with unknown fungal taxa and Sporormiaceae.

### Keystone taxa

Keystone taxa analysis (degree ≥15 and cluster coefficient >0.20) revealed 75, 69, and 68 keystone taxa for acidic, alkaline, and neutral soils, respectively. Bacterial phyla were the most common keystone taxa across all pH categories, while fungal keystone taxa were mostly found in neutral soils. The top four keystone taxa across all pH categories were Beijerinckiacea, Bacillaceae, Rubrobacteriaceae, and Pyrinomonadaceae. Family Sporormiaceae was the major fungal keystone taxa, and it was associated with neutral soils (1, 3 and 12 ASVs in acidic, alkaline, and neutral soils, respectively). At the family level, our analysis indicated distinct keystone taxa preference by pH. For example, the keystone taxa in acidic soils comprised Bryobacteraceae, Chthoniobacteraceae, Kineosporiaceae and Nocardioidaceae. The keystone taxa in alkaline soils included Chaetomiaceae, Cytophagaceae, Microbacteriaceae and Myxococcaceae. Oxalobacteraceae. Families Gaiellaceae, Micromonosporaceae, Streptomycetaceae and Aspergillaceae were identified as the keystone taxa in neutral soils. Some of the keystone taxa were shared in the three networks, including Acetobacteraceae, Bacillaceae, Beijerinckiaceae and Chitinophagaceae.

## DISCUSSION

This study provides a unique report of microbial diversity and its environmental controls in Sandveld, Hardveld and Wet‐sandveld ecological areas of Botswana on a landscape scale. Despite the extreme conditions in the study area, our analysis revealed high levels of edaphic microbial biodiversity, as shown by other studies in hyper‐arid environments (Andrew et al., 2012; Armstrong et al., 2016). At the phylum level, the bacterial and fungal composition between ecological zones was similar, with minor changes in the relative abundance. Members of the top 10 bacterial phyla identified in this study account for 92% of soil‐derived 16S rRNA and 16S rRNA gene libraries (Janssen, 2006), are ubiquitous in most soils (Delgado‐Baquerizo et al., 2018), and predominate in dryland soils (Coleine et al., 2024; McHugh et al., 2017; Wang et al., 2017). Our findings are consistent with local‐scale studies of the country (Elliott et al., 2014; Mhete et al., 2020), where Actinomycetota, Pseudomonadota, and Acidobacteria dominate bacterial communities. Fungal communities had a high relative abundance of Ascomycota. This phylum dominates biomes globally (Prober et al., 2015; Egidi et al., 2019a: Cowan et al., 2022), including drylands (Maestre et al., 2015). These results suggest that, at the phylum level, the soil microbiome of our study area is similar to other drylands.

The dominance of these specific phyla in dryland soils can be attributed to their diverse adaptations to extreme environmental stresses (Leung et al., 2020). For instance, members of Actinomycetota tolerate low moisture conditions, form stress‐resistant spores, and utilize atmospheric trace gases (H_2_ and CO) to maintain energy and fix CO_2_ into biomass in the dormant state (Bull et al., 2018; Leung et al., 2020; Lynch et al., 2014). Furthermore, members of this phylum are tolerant to UV radiation due to melanin production (Bull et al., 2018). Bacillota form heat‐resistant spores and can detect nutrient scarcity through chemotaxis (Filippidou et al., 2016; Makhalanyane et al., 2015). Conversely, the dominance of Ascomycota in drylands is thought to be due to the production of ultraviolent‐radiation‐resistant melanin (Challacombe et al., 2019; Egidi et al., 2019a). Variations in the soil properties of the different ecological zones were reflected by the significant clustering of microbial communities by ecological zones. The prevailing environmental conditions could have potentially selected specific microbial taxa for these ecological zones, which was reflected by the distinct indicator taxa observed in association with the ecological zones evaluated in this study. For fungal communities, Sandveld soils were populated by members of the order Filobasidiales, with a high abundance of the genus *Naganishia*. *Naganishia* species are typically desiccation‐tolerant, can withstand high levels of UV radiation (Pulschen et al., 2015), and populate oligotrophic environments (Schmidt et al., 2017). Taxa belonging to Pleosporales (*Teichospora*) were also associated with Sandveld soils. The dominance of these orders in harsh environments is attributed to darkly pigmented hyphae and melanised spores that may confer protection against desiccation and UV radiation (Bates et al., 2012). Similarly, Egidi et al. (2019b) identified Pleosporales as an indicator taxa for arid sites on a continental scale. *Brevibacillus*, an indicator bacterial taxa found in the Sandveld, survives in arid conditions through the production of exopolysaccharide (EPS) (Astorga‐Eló et al., 2021). Production of exopolysaccharides (EPS) by microorganisms confers resistance to ultraviolet radiation, extreme temperature, extreme pH, high salinity, high pressure, and poor nutrients (Yin et al., 2019).

The genus *Bryobacter*, an acidophile commonly isolated in acidic soils (Dedysh et al., 2017), was overrepresented in the Hardveld, possibly due to the low pH soils characteristic of this area. Hardveld soils were also uniquely associated with the orders Capnodiales and Hypocreales (*Bisifusarium*), whose members include plant pathogenic fungi infecting a broad range of hosts (Abdollahzadeh et al., 2020; Crous et al., 2009; Gryzenhout et al., 2017; Lombard et al., 2015). Hardveld zones have relatively fertile soils that make arable agriculture viable (Mphale et al., 2018). As a result, the association between Hardveld soils and pathogenic fungi may be due to the zone's high crop production and the dominance of tropical grassland. The abundance of Rokubacteriales in the Wet‐sandveld could have been enhanced by the presence of peatlands with high Ca and Mg levels, which are characteristic of this ecological zone (Ivanova et al., 2021). Members of Glomeromycota in the family Glomeraceae were indicator taxa for Wet‐sandveld soils. Glomeraceae promote plant growth by facilitating nutrient uptake and conferring plant tolerance to biotic and abiotic stress (Smith & Read, 2010). The association of these taxa with Wet‐sandveld soils may be related to the high plant species richness in Wet‐sandveld areas, as indicated by the high NPP in this ecological zone (Johnson et al., 2005). The most differentially abundant bacterial taxa for Sandveld ecozones were members of the Paenibacillales (*Paenibacillus*) and Brevibacillales (*Brevibacillus*). Members of these orders tolerate extremes of pH, temperature, salinity and drought (Yadav et al., 2015). Taxa belonging to Pleosporales (*Teichospora*) were also associated with Sandveld soils. Pleosporales are frequently found under extremely dry conditions (Bates et al., 2012; Knapp et al., 2015). Our results suggest that the growth of these taxa may be enhanced by the oligotrophic conditions in the Sandveld.

Our findings underscore the significance of edaphic and floristic variables (pH, NPP and Al, Mg, TOC, and Ca contents) in explaining variation in soil microbial diversity. We observed significantly lower soil microbial alpha diversity in arid Sandveld compared to Hardveld and Wet‐sandveld ecological areas. Our results suggest that microbial responses are driven by decreases in NPP and soil TOC content associated with increasing aridity (Berdugo et al., 2020; Maestre et al., 2015). NPP was the universal predictor of bacterial and fungal species richness in this study but is also a major driver of bacterial alpha diversity in global drylands (Delgado‐Baquerizo et al., 2018). NPP has an indirect effect on fungal diversity via its influence on other environmental variables such as soil TOC (Maestre et al., 2015; Yang et al., 2017). This supports our findings that show a strong positive correlation between fungal species richness and soil TOC. This finding also suggests that in low carbon drylands, microbial communities are limited by soil TOC (Delgado‐Baquerizo & Eldridge, 2019; Maestre et al., 2015). The significance of soil TOC in influencing microbial diversity is consistent with landscape‐scale patterns observed in drylands (Maestre et al., 2015; Zeng et al., 2019) and tussock grasslands (Egidi et al., 2019b).

Soil pH is generally regarded as a major predictor of bacterial richness across a wide range of systems (Cowan et al., 2022; Fierer et al., 2009; Lauber et al., 2009), although it does not have great importance in drylands where pH values are >6.5 (Maestre et al., 2015; Neilson et al., 2017; Vásquez‐Dean et al., 2020). Despite these contradictions, the strong relationships (positive or negative) between soil pH, bacterial richness and major bacterial and fungal phyla observed in this study support the notion that soil pH is one of the major drivers of changes in microbial community composition in the Botswana drylands (Bahram et al., 2018; Lauber et al., 2009).

Of the climatic variables (MAT and MAP) assessed in our study, MAP influenced bacterial and fungal community structure and relative abundance. Similarly, previous studies have identified climatic variables as drivers of soil community structure (Chen et al., 2017; McHugh et al., 2017; Vásquez‐Dean et al., 2020; Větrovský et al., 2019). The strong influence of MAP on dominant bacterial and fungal phyla was not surprising but is highly relevant as both factors will continue to be affected by climate change in arid environments (Fu & Feng, 2014; Knutti et al., 2008). Precipitation is fundamental for the functioning of arid and semi‐arid ecosystems and biodiversity regulation (Bai et al., 2008; Liu et al., 2009). Our results show that TOC, TN, and NPP positively correlate with MAP. Additionally, the driest ecological zone, Sandveld, was characterised by significantly low TOC, TN, and NPP and had the lowest species richness. This highlights the strong influence of a decrease in precipitation on microbial communities due to the unavailability of water‐soluble substrates or reduced soil nutrient availability and NPP (Bai et al., 2008; Schimel, 2018).

Various studies have highlighted the importance of environmental selection and dispersal limitation in structuring soil microbial communities in different environments (Wang et al., 2015, 2017; Zeng et al., 2019). Similarly, our results underscore the importance of these processes in structuring the biogeographic patterns of bacterial and fungal β‐diversity. However, the relative effects of these processes vary with taxa type. While some studies have shown that environmental factors are the primary drivers of variations in fungal communities (Chen et al., 2017), our findings suggest that variations in microbial community structure are influenced by both geographic distance and environmental heterogeneity at the landscape scale. A significant portion of the community variation (78% for bacteria and 86% for fungi) remained unexplained by variation partitioning analysis (VPA). Although widely used to determine variations in community structure, VPA may not be the most efficient method to determine the relative importance of environmental filtering and spatial limitation (Gilbert & Bennett, 2010; Smith & Lundholm, 2010). Additionally, variations in microbial communities among ecosystems are due to both deterministic (e.g., environmental filtering, plant diversity, and species interactions) and stochastic processes (e.g., dispersal limitation, speciation, and genetic drift) (Wang et al., 2013; Zhou & Ning, 2017). It has been shown that the soil microbial communities of Antarctica (McMurdo Dry Valleys) follow both stochastic and deterministic processes, but fine‐scale taxonomic resolution (i.e., identification to species level) is necessary to decipher such interactions (Lee et al., 2018). Therefore, unexplained variations may be due to some of these factors that were unaccounted for in this study. We suspect that both processes, mostly deterministic, also drive microbial communities in the Botswana drylands. However, further analysis is required to prove our hypothesis.

This study assessed the co‐occurrence patterns of bacteria and fungi in acidic, alkaline, and neutral soils, demonstrating that soil pH significantly impacts the potential relationships between soil taxa. Bacterial ASVs dominated the nodes across pH categories, corresponding with the higher bacterial richness observed in this study. Positive correlations were more common than negative correlations, suggesting the dominance of mutualistic and synergistic interactions. Negative interactions were mainly fungi–fungi interactions. Microorganisms that coexist in the environment compete for resources such as nutrients or space (Ghoul & Mitri, 2016). These findings suggest strong competitive exclusion between the different fungal species (Wardle et al., 1993) and narrow specialised niches in these soils (Yang et al., 2022). Furthermore, some microorganisms produce secondary metabolites in the soil that confer a competitive advantage (Keller et al., 2005). Our findings indicate that families involved in negative interactions include fungi Aspergillaceae and Trichocomacea. These families produce secondary metabolites such as polyketides and terpenoids, which have antifungal and antibacterial activities (Al‐Fakih & Almaqtri, 2019).

Soil bacteria and fungi may positively influence each other, and these interactions are critical for the proper functioning of an ecosystem (Deveau et al., 2018; Frey‐Klett et al., 2011). We observed high bacteria–fungi interactions in the neutral pH network. Bacteria thrive in alkaline soils, while fungi dominate acidic ones, making neutral soils conducive to synergistic interactions between bacteria and fungi. Additionally, the fungal species involved in the co‐occurrence interactions remained consistent across the analysed pH networks. However, the bacterial phyla participating in these interactions varied depending on the pH network being examined. The consistency of fungal species across the different pH networks suggests these taxa thrive in a broad range of pH levels compared to bacterial species (Rousk et al., 2010). Our findings underscore that variations in soil pH do not only influence the composition of bacterial species but also the specific interactions they form with fungal families. Microbial interactions are dependent on resource availability and environmental variables (Zheng et al., 2021). Soil nutrients in our study vary significantly according to the soil pH and may have influenced the observed microbial interactions (Table S8). For instance, concentrations of potassium, calcium and magnesium varied significantly across different pH categories, potentially resulting in variations in microbiota composition according to soil pH.

Our findings indicate that in acidic and neutral networks, the fungal families co‐occurred with the bacterial families primarily involved in phosphate solubilisation, nitrogen fixation, and decomposition of organic matter. These include bacterial families Bacillaceae, Paenibacillaceae, Gaiellaceae, and Roseiflexaceae (Mandic‐Mulec et al., 2016; Siddiqi et al., 2017; Thiel & Hanada, 2015; Wang et al., 2022). Fungi and bacteria interact in different environments, influencing carbon mineralisation and decomposition. Nonetheless, fungi are generally more efficient than bacteria in degrading polymers such as lignin and cellulose (Romaní et al., 2006), enabling bacteria to mineralize intermediate decomposition products released by fungi (Dang et al., 2021; Phukhamsakda et al., 2016; Romaní et al., 2006). The bacteria may also benefit from the interaction by directly assimilating the fungal decomposition products such as soluble sugars, amino acids and other metabolites. In return, the bacteria contribute to the pool of available phosphorus and nitrogen in the soil, which may provide the interacting fungi with a competitive advantage (Gómez‐Brandón et al., 2020). Bacterial metabolic byproducts may also create favourable microenvironments that may enhance fungal enzyme production necessary for further decomposition of organic material (Raczka et al., 2021).

In alkaline networks, bacteria–fungi interactions were dominated by bacterial families such as Chitinophagaceae and Roseiflexaceae which are involved in chitin and cellulose degradation and organic matter decomposition (Chung et al., 2012; Thiel & Hanada, 2015). However, this did not positively influence the carbon content of alkaline soils in this study, possibly due to reduced microbial activity and slower decomposition of organic matter due to high pH (Guo et al., 2023). Additionally, the high salinity of alkaline soils may have exacerbated the negative influence of high pH on microbial organic matter decomposition rates (Table S8). Organic matter may have been less available due to good aggregation in saline soils, or salinity‐induced stress may have reduced microbial efficiency in organic matter decomposition (Singh, 2016). Furthermore, these results suggest functional redundancy in carbon mineralisation in different soils owing to the contrasting influence of soil pH on microbial growth. This means that even if the pH negatively influences one group, the other group can compensate for it and maintain the overall function of carbon decomposition.

Microbial keystone taxa are crucial for the functioning of the soil ecosystem and determining community structure (Banerjee et al., 2018). We identified several keystone taxa that were also influenced by the soil pH. Furthermore, most of these taxa are potentially involved in organic matter decomposition. Keystone taxa known to degrade organic matter include Bacillaceae, Chitinophagaceae, Beijerinckiaceae and Sporormiaceae, observed across the three networks (Chiba et al., 2021; Lynd et al., 2002; Raczka et al., 2021; Sun et al., 2017; Trujillo et al., 2014). Different studies highlight the significance of keystone taxa in lignocellulose degradation, and these have been linked to enhanced decomposition efficiency and enzyme activity (Meng et al., 2022; Xiao et al., 2022; Zheng et al., 2021). Nonetheless, these interactions may also indicate a possible functional redundancy in these communities (Banerjee et al., 2018). In addition, members of the family Bacillaceae are found in diverse habitats and play key roles in denitrification, nitrogen fixation, and phosphate solubilisation (Mandic‐Mulec et al., 2016). Nocardioidaceae break down recalcitrant chemicals and are involved in the bioremediation of environmental pollutants.

## CONCLUSION

This is the first study to investigate soil bacterial and fungal communities, using high throughput sequencing technology, at the landscape scale in Botswana. Our findings show that microbial community structures differ by ecological zones, with Sandveld soils having significantly lower alpha diversity than Wet‐sandveld and Hardveld. Nonetheless, the major phyla observed across the ecological zones corroborate the findings of other drylands, such as the Namib and Atacama deserts. The most important driver of microbial diversity and composition is soil pH. Climatic variables (MAP and MAT) significantly correlated with community diversity. Furthermore, our results indicated that environmental filtering played a significant role in bacterial community variation while geographic distance influenced fungal communities. Our findings close a knowledge gap in the biogeographic patterns of Botswana soils and provide insights into how soil microbial communities may respond to environmental changes. Although the Sandveld, Wet‐sandveld and Hardveld ecological zones of Botswana harbor diverse microorganisms, this study nevertheless represents only a baseline for future research, where issues such as functional diversity, inter‐taxon interactions and community responses to climate change remain unexplored.

## AUTHOR CONTRIBUTIONS

**Coetzee Tidimalo:** Conceptualization; data curation; formal analysis; investigation; methodology; software; validation; visualization; writing – original draft; writing – review and editing. **Ortiz Maximiliano:** Data curation; formal analysis; methodology; supervision; writing – review and editing. **Jordaan Karen:** Formal analysis; methodology; software; visualization; writing – review and editing. **Pedro H. Lebre:** Data curation; formal analysis; methodology; visualization; writing – review and editing; supervision. **Olivier Bernard:** Data curation; formal analysis. **Greve Michelle:** Formal analysis; writing – review and editing. **Dikinya Oagile:** Supervision; writing – review and editing. **Don A. Cowan:** Conceptualization; project administration; resources; funding acquisition; writing – review and editing; supervision.

## CONFLICT OF INTEREST STATEMENT

The authors declare no conflicts of interest.

## Supporting information


**FIGURE S1:** Relative abundance of the major (A) bacterial and (B) fungal phyla (≥5%) in Hardveld, Sandveld, and Wet‐sandveld ecological zones.
**FIGURE S2:** Relationship between the predictor variables from the best subset model and microbial richness: bacterial (A–C) and fungal (D–F) across different ecological zones.
**FIGURE S3:** The correlation analysis between environmental variables and dominant (A) bacterial and (B) fungal relative abundances at the phylum level. Blue and red represent positive and negative correlations, respectively.
**FIGURE S4:** Partial residual plots showing the relationship between Bray–Curtis dissimilarity and the geographic distance of (A) bacterial and (B) fungal communities. The *p* values indicate the significance of geographic distance.
**FIGURE S5:** Plot of LDA scores of differentially abundant taxa in (A) bacterial communities and (B) fungal communities.
**FIGURE S6:** Co‐occurrence incidence of bacteria–bacteria, bacteria–fungi, and fungi–fungi interactions across the different pH networks.
**FIGURE S7:** Co‐occurrence network clusters of bacterial and fungal communities in (A) acidic, (B) alkaline, and (C) neutral soils.

**TABLE S1:** Physicochemical variables from the topsoil (5 m depth) obtained from the 89 sampling sites across the Hardveld, Sandveld, and Wet_sandveld ecological zones.

**TABLE S2:** The correlation matrix between environmental variables. Values represent the Spearman's correlation coefficient.
**TABLE S3:** Final selected best subset regression model of the predictors of bacterial species alpha diversity (observed) in the different ecological regions.
**TABLE S4:** Final selected best subset regression model of the predictors of fungal species alpha diversity (observed) in the different ecological regions.
**TABLE S5:** Summary of the redundancy analysis (RDA) results showing significant variables influencing bacterial community structure and diversity in the 89 sampling sites.
**TABLE S6:** Summary of the redundancy analysis (RDA) results showing significant variables influencing fungal community structure and diversity in the 89 sampling sites.
**TABLE S7:** Topological properties of the network of the acidic, alkaline, and neutral networks.
**TABLE S8:** Mean values for the environmental variables for the soils across the different pH categories.

## Data Availability

The data that support the findings of this study are openly available in SRA NCBI at https://www.ncbi.nlm.nih.gov, reference number PRJNA807934.
