# Supplementary material for: Microbial diversity in the arid and semi‐arid soils of Botswana
Source: Environ Microbiol Rep. 2024 Nov 13;16(6):e70044. doi: 10.1111/1758-2229.70044 (PMC11558117; doi:10.1111/1758-2229.70044)
Supplement: Supplementary file 1 — FIGURE S1: Relative abundance of the major (A) bacterial and (B) fungal phyla (≥5%) in Hardveld, Sandveld, and Wet‐sandveld ecological zones. FIGURE S2: Relationship between the predictor variables from the best subset model and microbial richness: bacterial (A–C) and fungal (D–F) across different ecological zones. FIGURE S3: The correlation analysis between environmental variables and dominant (A) bacterial and (B) fungal relative abundances at the phylum level. Blue and red represent positive and negative correlations, respectively. FIGURE S4: Partial residual plots showing the relationship between Bray–Curtis dissimilarity and the geographic distance of (A) bacterial and (B) fungal communities. The p values indicate the significance of geographic distance. FIGURE S5: Plot of LDA scores of differentially abundant taxa in (A) bacterial communities and (B) fungal communities. FIGURE S6: Co‐occurrence incidence of bacteria–bacteria, bacteria–fungi, and fungi–fungi interactions across the different pH networks. FIGURE S7: Co‐occurrence network clusters of bacterial and fungal communities in (A) acidic, (B) alkaline, and (C) neutral soils. [file EMI4-16-e70044-s003.docx]

**Supplementary Information**


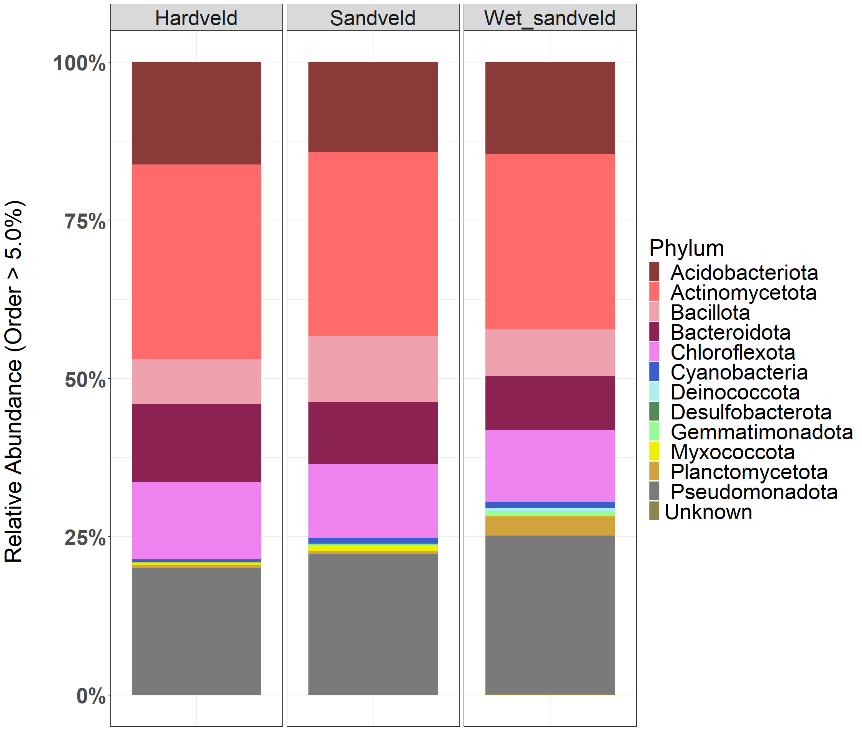


**Fig. S1A**


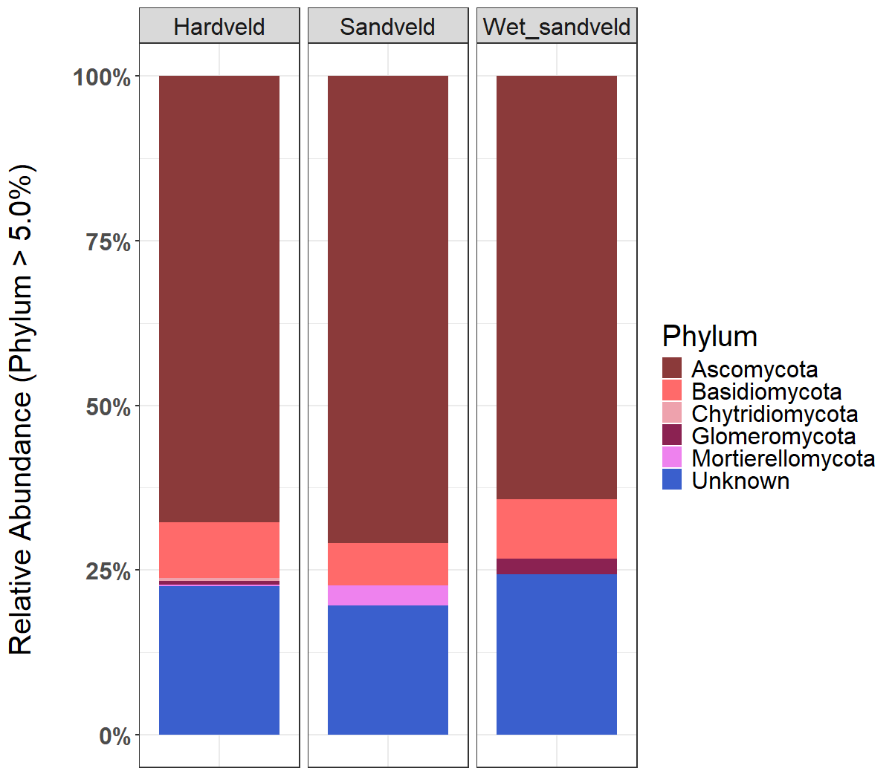


**Fig. S1B**

**Figure S1**: Relative abundance of the major (**A**) bacterial and (**B**) fungal phyla (≥5%) in Hardveld, Sandveld, and Wet-sandveld ecological zones.


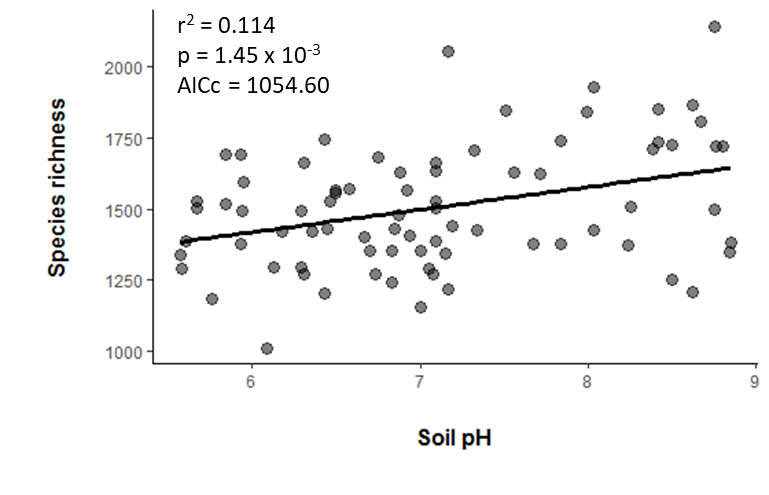


**Fig. S2A**


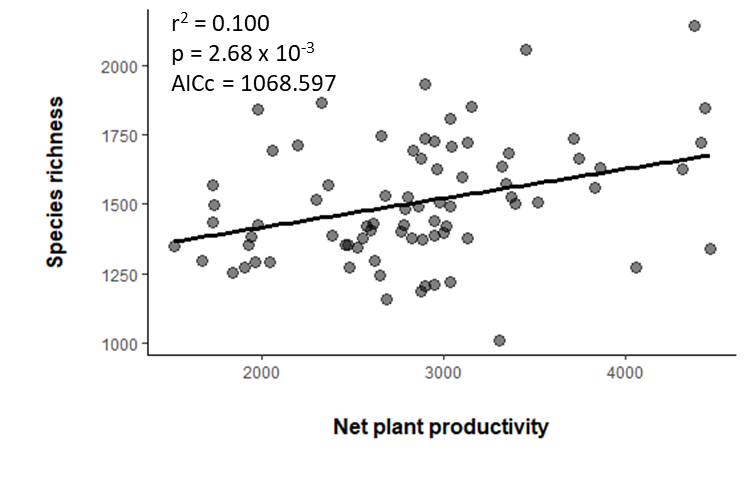


**Fig. S2B**

**
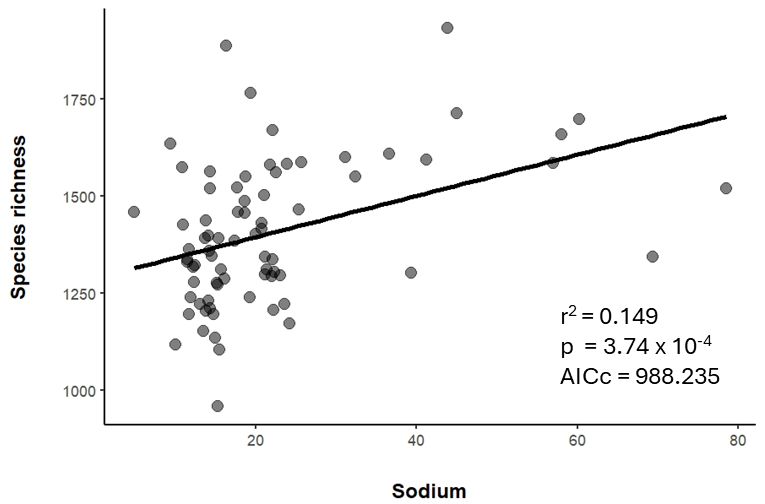
**

**Fig. S2C**

**
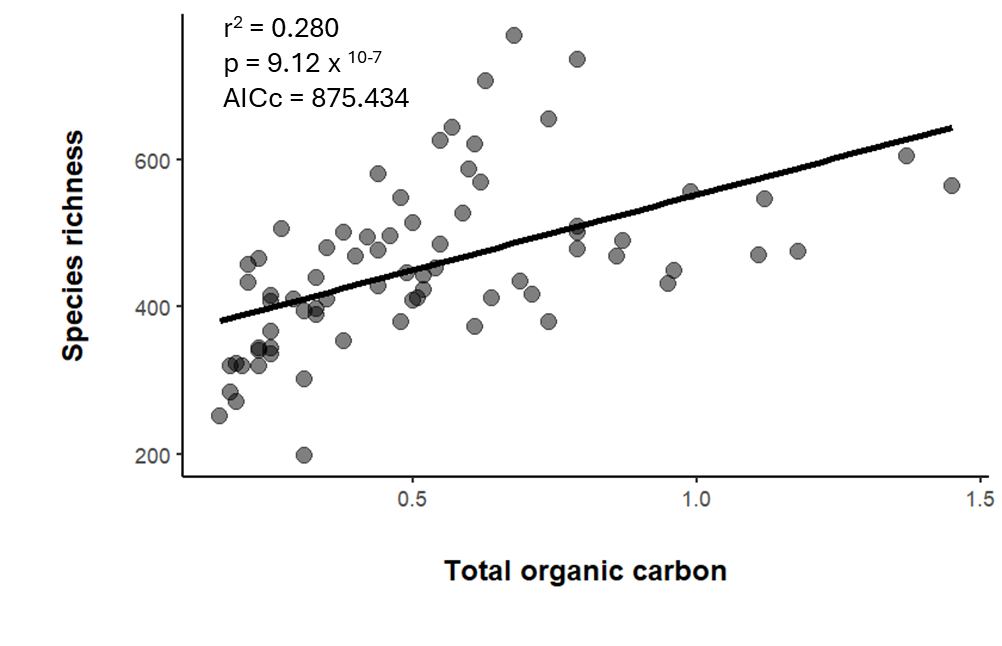
**

**Fig. S2D**

**
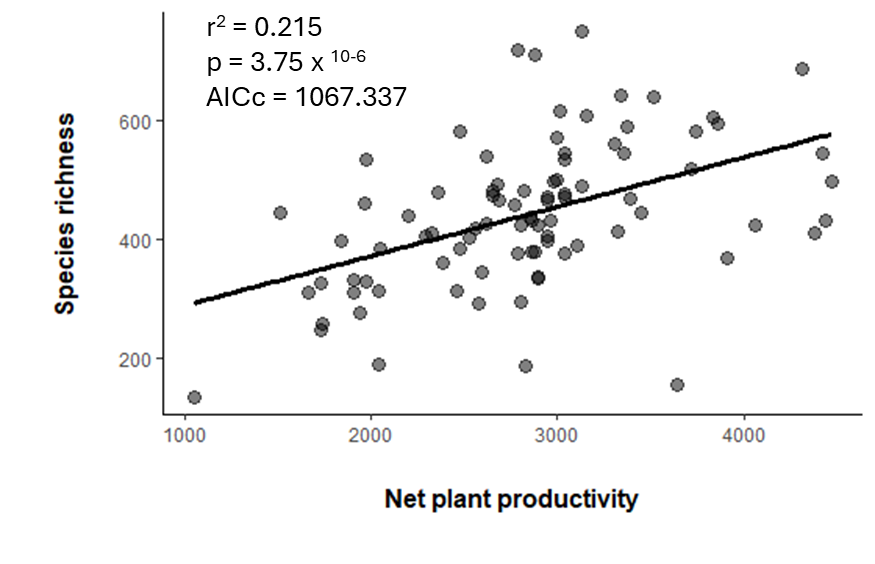
**

**Fig. S2E**

**
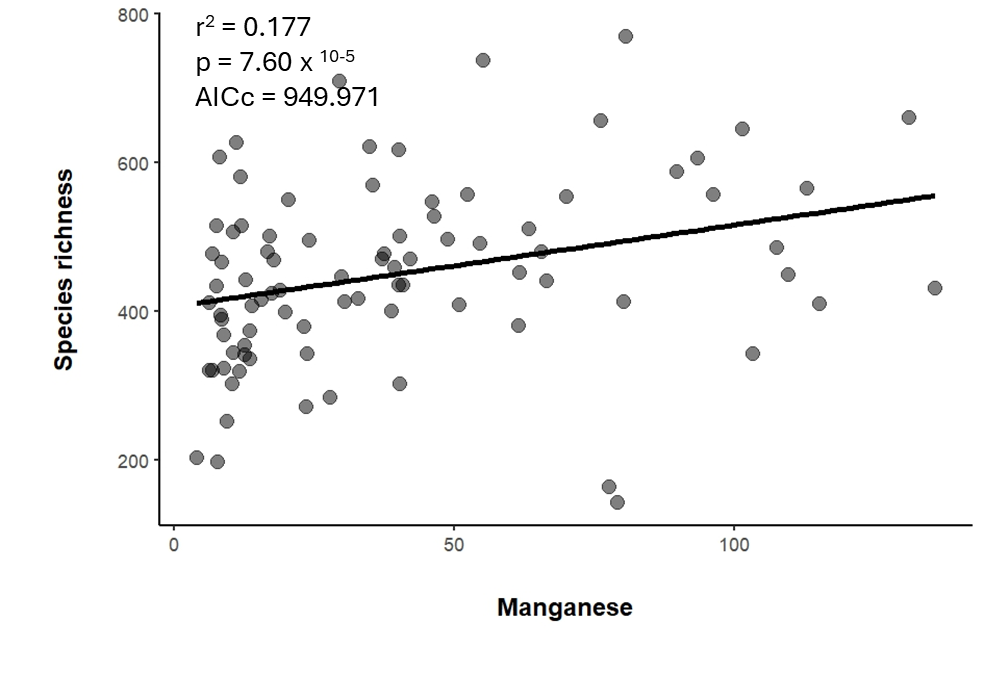
**

**Fig. S2F**

**Figure S2**: Relationship between the predictor variables from the Best subset model and microbial richness: bacterial (**A-C**) and fungal (**D-F**) across different ecological zones.


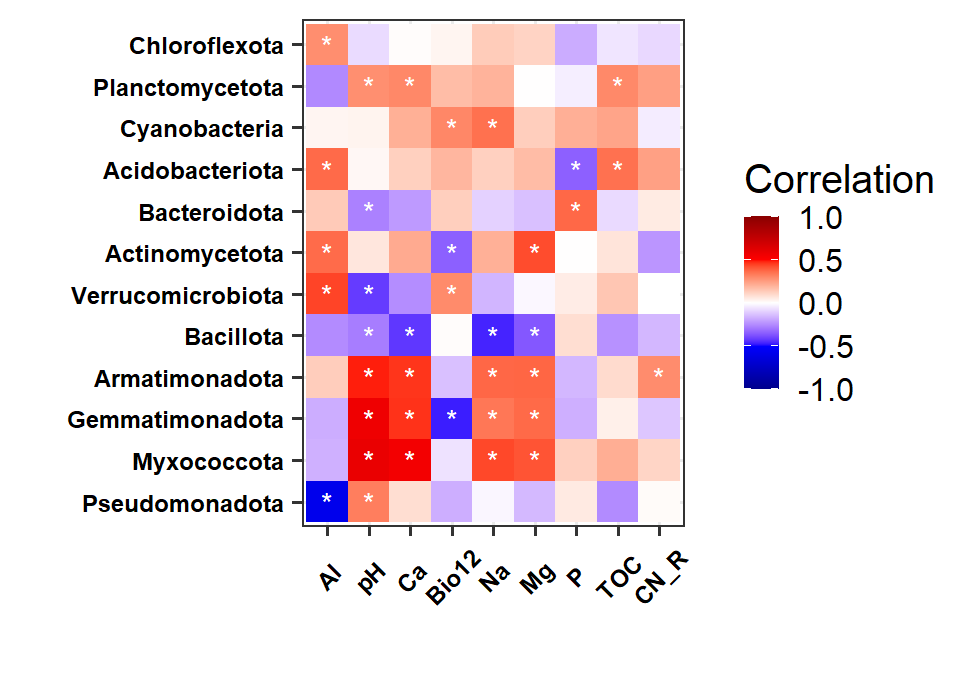


**Fig. S3A**


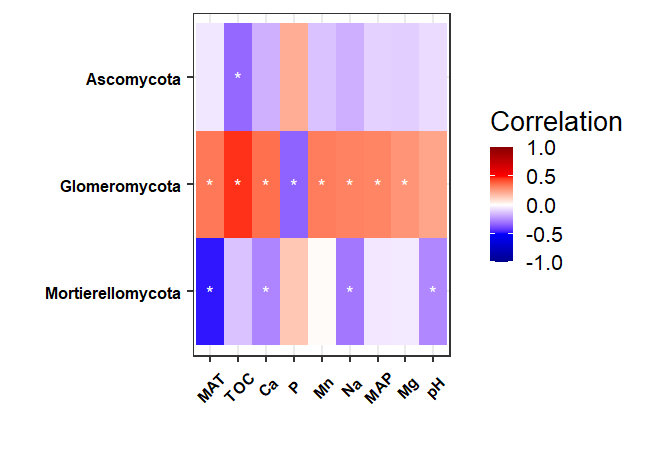


**Fig. S3B**

**Figure S3:** The correlation analysis between environmental variables and dominant (**A**) bacterial and (**B**) fungal relative abundances at the phylum level. Blue and red represent positive and negative correlations, respectively.

**
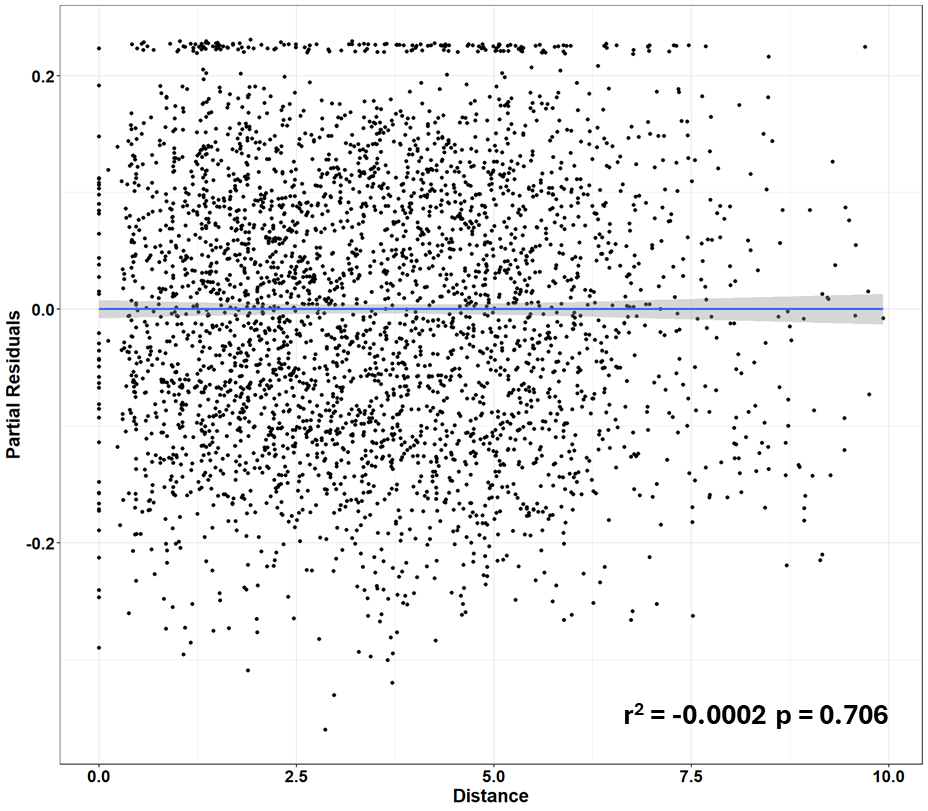
**

**Fig. S4A**


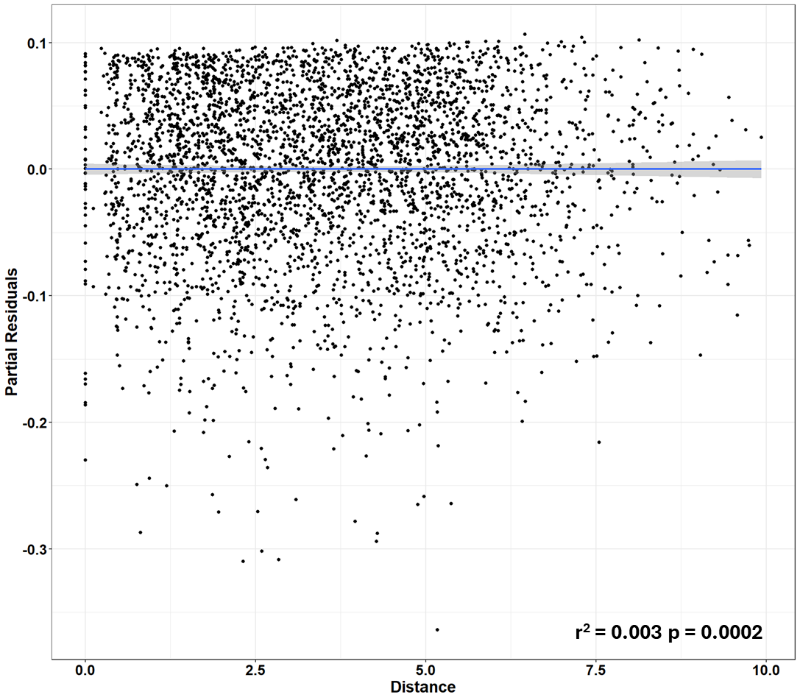


**Fig. S4B**

**Figure S4**. Partial residual plots showing the relationship between Bray-Curtis dissimilarity and the geographic distance of A) bacterial and (B) fungal communities. The p values indicate the significance of geographic distance.

6
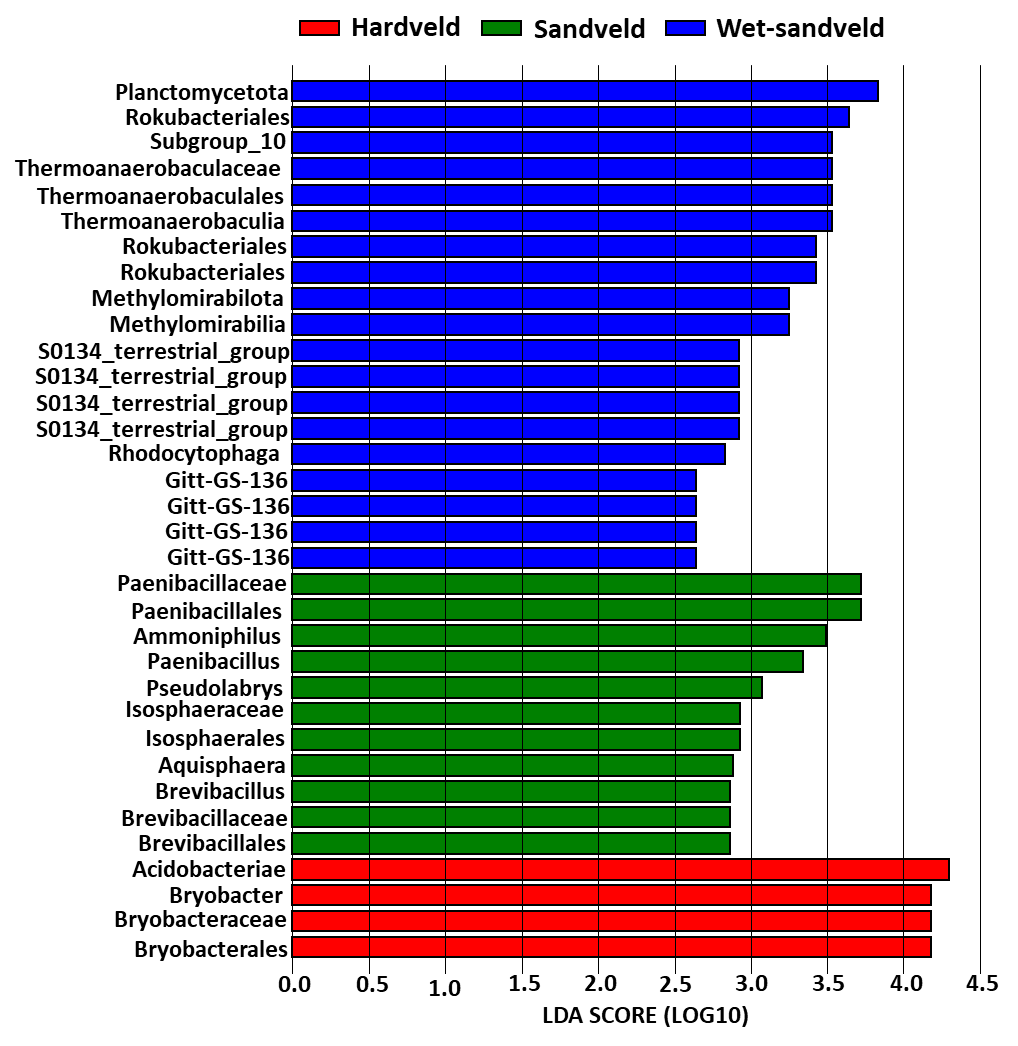


**Fig. S5A**

**
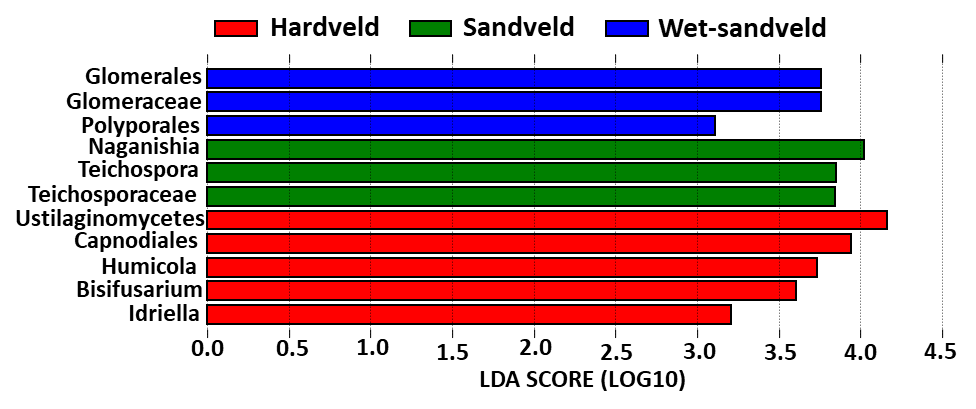
**

**Fig. S5B**

**Figure S5**: Plot of LDA scores of differentially abundant taxa in **A)** Bacterial communities and **B)** Fungal communities.

**Figure S6:** Co-occurrence incidence of bacteria-bacteria, bacteria-fungi, and fungi-fungi interactions across the different pH networks.


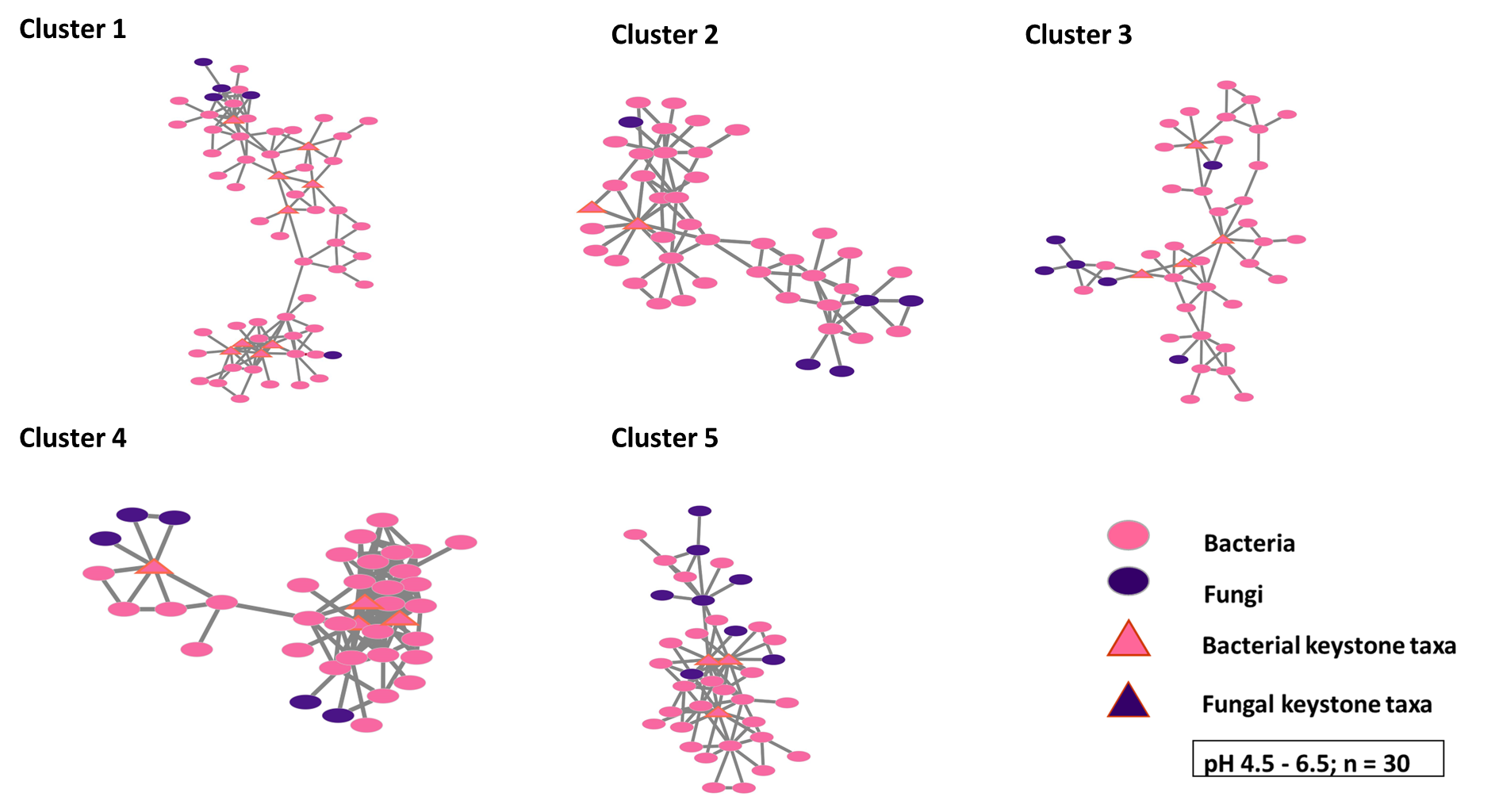


**Fig. S7A**

**
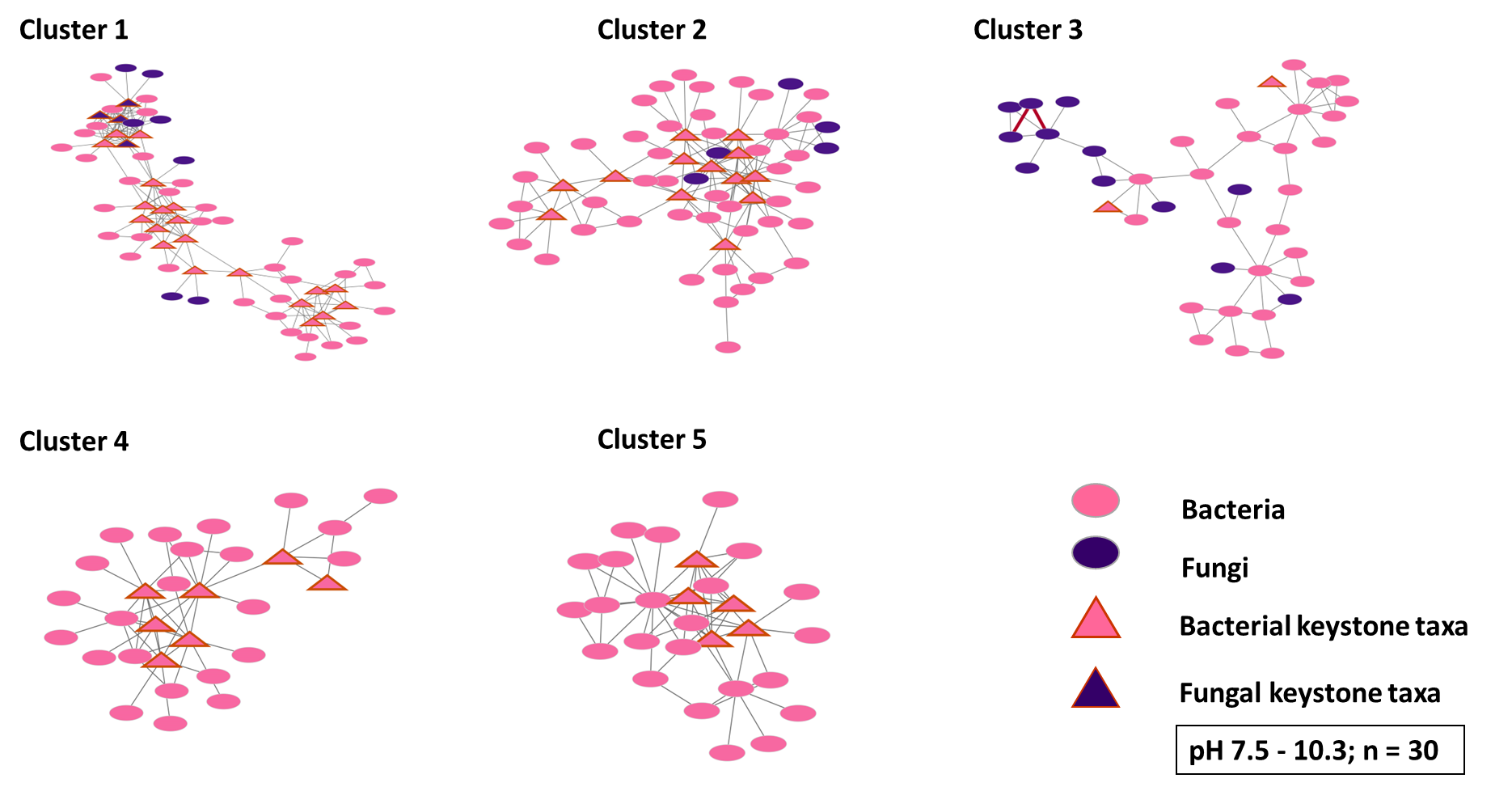
**

**Fig. S7B**

**
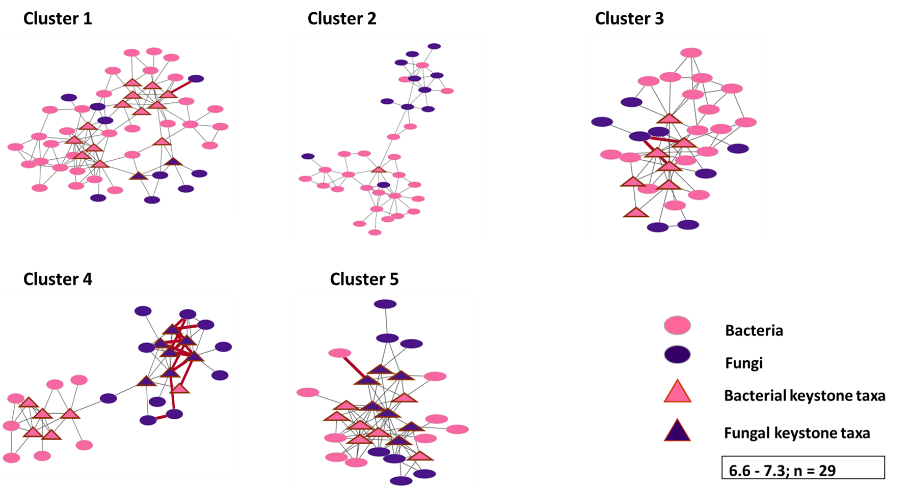
**

**Fig. S7C**

**Figure S7**: Co-occurrence network clusters of bacterial and fungal communities in A) acidic, B) alkaline, and C) neutral soils.
