# Supplementary material for: Microbial diversity in the arid and semi‐arid soils of Botswana
Source: Environ Microbiol Rep. 2024 Nov 13;16(6):e70044. doi: 10.1111/1758-2229.70044 (PMC11558117; doi:10.1111/1758-2229.70044)
Supplement: Supplementary file 2 — TABLE S1: Physicochemical variables from the topsoil (5 m depth) obtained from the 89 sampling sites across the Hardveld, Sandveld, and Wet_sandveld ecological zones. [file EMI4-16-e70044-s002.docx]

**Table S1.**  Physicochemical variables from the topsoil (5 m depth) obtained from the 89 sampling sites across the Hardveld, Sandveld and Wet_sandveld ecological zones.

| SampleID | pH | CN_R | TN  % | TOC % | K  mg/kg | Ca mg/kg | Mg mg/kg | Na mg/kg | P mg/kg | Mn mg/kg | Al mg/kg | Fe mg/kg | Sand % | Silt % | Clay  % |
| --- | --- | --- | --- | --- | --- | --- | --- | --- | --- | --- | --- | --- | --- | --- | --- |
| BW01 | 5.84 | 11.00 | 0.03 | 0.33 | 47.45 | 141.38 | 35.15 | 14.30 | 8.14 | 19.94 | 225.12 | 29.68 | 90 | 0 | 10 |
| BW02 | 5.84 | 14.50 | 0.02 | 0.29 | 26.44 | 86.89 | 33.46 | 13.70 | 6.15 | 6.33 | 209.88 | 21.04 | 90 | 0 | 10 |
| BW03 | 7.84 | 10.44 | 0.09 | 0.94 | 331.87 | 4305.84 | 215.57 | 36.65 | 50.05 | 103.30 | 290.09 | 86.64 | 77 | 3 | 20 |
| BW04 | 5.67 | 21.50 | 0.04 | 0.86 | 48.34 | 238.69 | 39.23 | 15.37 | 16.58 | 42.13 | 356.97 | 51.71 | 83 | 1 | 16 |
| BW05 | 6.88 | 10.00 | 0.25 | 2.50 | 623.41 | 2972.35 | 373.63 | 32.39 | 18.48 | 40.94 | 522.21 | 270.53 | 55 | 9 | 36 |
| BW06 | 7.34 | 12.00 | 0.08 | 0.96 | 411.93 | 1573.17 | 505.93 | 29.71 | 13.14 | 109.62 | 444.90 | 68.25 | 73 | 1 | 26 |
| BW07 | 7.08 | 12.00 | 0.05 | 0.60 | 105.08 | 788.46 | 142.89 | 22.23 | 10.03 | 89.73 | 346.85 | 39.39 | 85 | 0 | 15 |
| BW08 | 6.85 | 9.86 | 0.22 | 2.17 | 291.47 | 3611.34 | 1164.75 | 69.34 | 24.71 | 96.32 | 336.78 | 129.55 | 50 | 12 | 38 |
| BW09 | 6.09 | 12.40 | 0.05 | 0.62 | 153.74 | 263.83 | 78.56 | 15.32 | 18.72 | 35.47 | 320.83 | 41.13 | 89 | 0 | 11 |
| BW10 | 5.95 | 12.75 | 0.04 | 0.51 | 132.22 | 393.90 | 135.38 | 18.68 | 18.76 | 80.20 | 584.22 | 55.68 | 75 | 3 | 22 |
| BW11 | 4.51 | 12.58 | 0.12 | 1.51 | 283.37 | 673.79 | 236.85 | 38.33 | 44.17 | 38.90 | 925.34 | 234.85 | 48 | 9 | 43 |
| BW12 | 6.75 | 12.00 | 0.08 | 0.96 | 155.81 | 1252.16 | 163.88 | 21.75 | 60.37 | 70.09 | 294.09 | 78.37 | 72 | 1 | 26 |
| BW13 | 6.58 | 12.33 | 0.06 | 0.74 | 117.49 | 753.65 | 209.82 | 18.69 | 9.60 | 76.17 | 485.45 | 57.45 | 77 | 5 | 18 |
| BW14 | 6.46 | 15.80 | 0.05 | 0.79 | 70.36 | 506.01 | 90.48 | 13.86 | 27.61 | 40.26 | 297.36 | 65.78 | 86 | 0 | 14 |
| BW15 | 6.5 | 15.33 | 0.03 | 0.46 | 59.96 | 1015.65 | 230.15 | 25.38 | 5.99 | 48.88 | 402.96 | 70.33 | 78 | 0 | 22 |
| BW16 | 7.09 | 13.17 | 0.06 | 0.79 | 128.71 | 1934.46 | 1048.74 | 85.05 | 10.37 | 63.40 | 364.25 | 46.64 | 60 | 6 | 35 |
| BW17 | 6.29 | 14.14 | 0.07 | 0.99 | 62.87 | 531.18 | 107.95 | 14.10 | 11.89 | 52.36 | 277.24 | 51.9 | 86 | 0 | 14 |
| BW18 | 7.09 | 12.45 | 0.11 | 1.37 | 162.37 | 1593.74 | 229.92 | 20.72 | 24.64 | 93.42 | 366.29 | 65.64 | 80 | 1 | 19 |
| BW19 | 7.09 | 11.89 | 0.09 | 1.07 | 161.06 | 1267.17 | 242.49 | 18.74 | 4.29 | 204.55 | 429.60 | 36.34 | 77 | 1 | 22 |
| BW20 | 8.03 | 13.17 | 0.06 | 0.79 | 191.20 | 2343.09 | 190.97 | 22.13 | 5.11 | 55.28 | 340.57 | 47.28 | 84 | 0 | 16 |
| BW21 | 7.67 | 13.60 | 0.05 | 0.68 | 178.09 | 1638.21 | 170.08 | 22.30 | 5.36 | 80.58 | 378.61 | 41.05 | 84 | 0 | 16 |
| BW22 | 7.19 | 16.67 | 0.03 | 0.50 | 69.94 | 840.92 | 119.53 | 14.28 | 5.62 | 51.01 | 271.41 | 38.14 | 88 | 0 | 12 |
| BW23 | 6.43 | 19.00 | 0.02 | 0.38 | 21.79 | 219.36 | 41.57 | 9.37 | 1.82 | 17.07 | 220.96 | 23.45 | 90 | 0 | 10 |
| BW24 | 8.25 | 10.75 | 0.08 | 0.86 | 349.38 | 8576.32 | 1144.20 | 77.64 | 5.78 | 131.11 | 347.29 | 40.5 | 53 | 3 | 44 |
| BW25 | 5.57 | 25.00 | 0.02 | 0.50 | 43.52 | 131.23 | 35.29 | 11.93 | 6.82 | 7.72 | 239.08 | 47.21 | 90 | 0 | 10 |
| BW26 | 7.17 | 14.50 | 0.10 | 1.45 | 137.98 | 2114.07 | 199.69 | 24.18 | 9.20 | 112.88 | 372.93 | 36.35 | 81 | 0 | 19 |
| BW27 | 6.36 | 14.25 | 0.04 | 0.57 | 141.77 | 641.00 | 245.62 | 21.22 | 7.90 | 101.39 | 452.52 | 58.59 | 79 | 0 | 21 |
| BW28 | 7.32 | 12.43 | 0.07 | 0.87 | 183.23 | 4450.31 | 604.97 | 41.25 | 4.67 | 54.55 | 565.00 | 48.88 | 55 | 8 | 37 |
| BW29 | 10.28 | 18.00 | 0.03 | 0.54 | 1025.07 | 8867.28 | 138.84 | 3833.51 | 0.47 | 12.10 | 0.00 | 11.42 | 80 | 0 | 20 |
| BW30 | 8.42 | 11.91 | 0.11 | 1.31 | 149.98 | 7692.63 | 176.42 | 43.81 | 4.91 | 40.12 | 0.00 | 12.33 | 77 | 9 | 14 |
| BW31 | 8.80 | 27.00 | 0.01 | 0.27 | 28.94 | 3442.37 | 86.41 | 22.50 | 5.01 | 10.70 | 87.80 | 20.62 | 89 | 0 | 11 |
| BW32 | 8.76 | 16.00 | 0.03 | 0.48 | 34.64 | 4768.29 | 111.00 | 25.65 | 5.20 | 20.51 | 1.81 | 22.97 | 90 | 0 | 10 |
| BW33 | 7.99 | 13.20 | 0.05 | 0.66 | 338.72 | 4370.18 | 2264.92 | 131.05 | 16.72 | 77.60 | 962.30 | 55.40 | 14 | 8 | 78 |
| BW34 | 8.58 | 13.43 | 0.07 | 0.94 | 372.78 | 6011.92 | 1902.24 | 132.30 | 3.97 | 79.07 | 456.85 | 46.66 | 29 | 7 | 63 |
| BW35 | 7.71 | 21.00 | 0.03 | 0.63 | 17.72 | 1213.45 | 49.52 | 17.69 | 9.11 | 29.55 | 169.13 | 29.43 | 91 | 0 | 9 |
| BW36 | 6.50 | 27.50 | 0.02 | 0.55 | 23.81 | 226.93 | 37.35 | 17.84 | 8.53 | 11.10 | 195.07 | 29.99 | 91 | 0 | 9 |
| BW37 | 8.75 | 14.20 | 0.05 | 0.71 | 47.05 | 4977.22 | 129.96 | 43.81 | 3.46 | 32.88 | 75.76 | 30.90 | 84 | 0 | 16 |
| BW38 | 8.62 | 21.33 | 0.03 | 0.64 | 103.39 | 4274.63 | 190.15 | 44.97 | 6.31 | 30.40 | 222.83 | 18.47 | 89 | 0 | 11 |
| BW39 | 8.50 | 11.29 | 0.07 | 0.79 | 190.29 | 4798.37 | 389.32 | 57.01 | 7.61 | 65.63 | 314.57 | 41.40 | 79 | 5 | 16 |
| BW40 | 8.03 | 22.00 | 0.02 | 0.44 | 37.51 | 664.71 | 113.82 | 19.39 | 4.48 | 18.91 | 129.91 | 34.44 | 92 | 0 | 8 |
| BW41 | 8.42 | 14.75 | 0.04 | 0.59 | 28.41 | 1722.77 | 134.28 | 31.11 | 8.63 | 46.51 | 99.14 | 29.10 | 92 | 0 | 8 |
| BW42 | 7.51 | 17.33 | 0.03 | 0.52 | 58.94 | 612.28 | 110.22 | 22.07 | 5.97 | 12.84 | 128.73 | 27.04 | 95 | 0 | 5 |
| BW43 | 6.31 | 11.65 | 0.26 | 3.03 | 248.60 | 2314.24 | 317.98 | 78.47 | 5.65 | 8.28 | 225.73 | 95.03 | 70 | 7 | 23 |
| BW44 | 7.17 | 10.00 | 0.04 | 0.4 | 54.00 | 533.35 | 109.79 | 16.36 | 4.47 | 17.86 | 126.51 | 31.42 | 94 | 0 | 6 |
| BW45 | 8.38 | 9.80 | 0.05 | 0.49 | 97.75 | 1080.50 | 146.69 | 23.85 | 10.85 | 29.86 | 129.83 | 20.23 | 93 | 0 | 7 |
| BW46 | 7.99 | 9.33 | 0.12 | 1.12 | 255.29 | 3730.70 | 255.96 | 60.28 | 12.60 | 46.04 | 315.88 | 35.32 | 66 | 10 | 24 |
| BW47 | 8.67 | 10.5 | 0.04 | 0.42 | 114.91 | 5138.30 | 104.28 | 58.01 | 4.91 | 24.12 | 0.00 | 16.63 | 92 | 0 | 8 |
| BW48 | 7.56 | 10.17 | 0.06 | 0.61 | 160.62 | 1116.17 | 415.20 | 21.08 | 7.39 | 34.89 | 218.41 | 30.97 | 85 | 0 | 15 |
| BW49 | 7.09 | 10.40 | 0.05 | 0.52 | 141.00 | 599.99 | 112.77 | 14.32 | 22.43 | 17.45 | 79.24 | 58.57 | 90 | 0 | 10 |
| BW50 | 6.13 | 1.39 | 0.44 | 0.61 | 1040.43 | 5575.66 | 920.01 | 131.82 | 21.45 | 40.29 | 234.82 | 540.46 | 27 | 51 | 22 |
| BW51 | 6.87 | 9.60 | 0.05 | 0.48 | 81.68 | 693.60 | 92.78 | 17.38 | 6.99 | 23.23 | 112.33 | 36.23 | 89 | 1 | 10 |
| BW52 | 8.24 | 9.70 | 0.1 | 0.97 | 144.20 | 7321.62 | 260.64 | 39.36 | 23.87 | 59.91 | 0.95 | 20.10 | 86 | 2 | 12 |
| BW53 | 6.73 | 9.86 | 0.07 | 0.69 | 23.08 | 596.11 | 47.71 | 13.09 | 16.20 | 40.08 | 121.60 | 27.35 | 94 | 0 | 6 |
| BW54 | 5.98 | 14.67 | 0.03 | 0.44 | 13.09 | 265.24 | 32.34 | 11.34 | 5.17 | 11.92 | 104.92 | 26.84 | 95 | 0 | 5 |
| BW55 | 5.96 | 15.25 | 0.04 | 0.61 | 7.52 | 245.08 | 20.84 | 6.28 | 5.12 | 13.68 | 75.87 | 16.46 | 96 | 0 | 4 |
| BW56 | 8.62 | 11.67 | 0.03 | 0.35 | 57.70 | 1700.06 | 106.27 | 15.01 | 10.58 | 115.14 | 120.45 | 35.10 | 90 | 0 | 10 |
| BW57 | 8.15 | 9.25 | 0.08 | 0.74 | 227.99 | 2951.62 | 250.52 | 21.23 | 16.00 | 61.49 | 319.61 | 42.51 | 85 | 0 | 15 |
| BW58 | 7.00 | 13.88 | 0.08 | 1.11 | 139.67 | 1397.76 | 154.82 | 15.50 | 10.80 | 37.16 | 244.98 | 49.67 | 89 | 0 | 11 |
| BW59 | 6.83 | 11.67 | 0.03 | 0.35 | 43.54 | 462.39 | 48.23 | 11.76 | 3.77 | 16.73 | 240.84 | 30.56 | 94 | 0 | 6 |
| BW60 | 6.94 | 12.67 | 0.03 | 0.38 | 67.47 | 494.45 | 100.84 | 12.46 | 6.18 | 12.64 | 137.31 | 28.70 | 95 | 0 | 5 |
| BW61 | 6.83 | 11.5 | 0.02 | 0.23 | 41.75 | 368.95 | 94.55 | 12.35 | 3.65 | 11.77 | 139.53 | 26.49 | 95 | 0 | 5 |
| BW62 | 6.43 | 11.5 | 0.02 | 0.23 | 44.00 | 262.81 | 71.08 | 13.49 | 6.10 | 12.67 | 146.65 | 25.28 | 95 | 0 | 5 |
| BW63 | 7.15 | 8.33333 | 0.03 | 0.25 | 51.55 | 546.76 | 131.46 | 15.19 | 6.13 | 15.61 | 172.35 | 29.81 | 92 | 0 | 8 |
| BW64 | 5.76 | 11 | 0.03 | 0.33 | 38.62 | 201.41 | 49.52 | 10.00 | 12.09 | 8.64 | 143.51 | 24.02 | 93 | 0 | 7 |
| BW65 | 6.7 | 10.3333 | 0.03 | 0.31 | 61.21 | 402.72 | 87.24 | 15.29 | 9.47 | 8.46 | 181.67 | 31.27 | 94 | 0 | 6 |
| BW66 | 6.67 | 11.5 | 0.02 | 0.23 | 57.01 | 324.27 | 87.06 | 11.50 | 7.91 | 8.66 | 200.20 | 28.56 | 94 | 0 | 6 |
| BW67 | 10.06 | 16.25 | 0.04 | 0.65 | 1677.33 | 4897.71 | 137.93 | 15400.64 | 37.07 | 4.02 | 0.00 | 8.95 | 70 | 0 | 30 |
| BW68 | 7.05 | 9.5 | 0.02 | 0.19 | 43.53 | 283.75 | 72.69 | 23.60 | 7.20 | 8.87 | 186.79 | 24.40 | 94 | 0 | 6 |
| BW69 | 7.09 | 12.5 | 0.02 | 0.25 | 54.75 | 368.61 | 96.14 | 15.69 | 4.83 | 8.95 | 209.77 | 27.45 | 93 | 0 | 7 |
| BW70 | 7.34 | 11.5 | 0.02 | 0.23 | 45.46 | 349.46 | 78.72 | 14.52 | 4.20 | 23.83 | 190.49 | 27.27 | 95 | 0 | 5 |
| BW71 | 6.31 | 12.5 | 0.02 | 0.25 | 50.64 | 252.16 | 81.95 | 14.33 | 5.85 | 10.63 | 281.85 | 36.54 | 92 | 0 | 8 |
| BW72 | 6.29 | 9 | 0.02 | 0.18 | 31.52 | 181.14 | 43.16 | 14.10 | 8.52 | 6.37 | 162.19 | 32.47 | 95 | 0 | 5 |
| BW73 | 6.45 | 12.5 | 0.02 | 0.25 | 32.79 | 302.51 | 51.62 | 11.69 | 14.20 | 13.66 | 194.06 | 25.85 | 94 | 0 | 6 |
| BW74 | 7.02 | 20 | 0.01 | 0.2 | 15.93 | 226.86 | 61.98 | 9.74 | 9.76 | 6.82 | 174.17 | 24.08 | 93 | 0 | 7 |
| BW75 | 8.5 | 12.5 | 0.02 | 0.25 | 59.39 | 781.23 | 120.00 | 14.73 | 14.27 | 13.92 | 138.73 | 23.67 | 96 | 0 | 4 |
| BW76 | 6.92 | 16 | 0.01 | 0.16 | 26.24 | 139.10 | 34.35 | 4.90 | 12.58 | 9.49 | 139.31 | 13.57 | 95 | 0 | 5 |
| BW77 | 8.84 | 5.25 | 0.04 | 0.21 | 72.23 | 2390.79 | 190.18 | 16.14 | 34.27 | 39.39 | 135.56 | 37.46 | 91 | 0 | 9 |
| BW78 | 8.85 | 18 | 0.01 | 0.18 | 77.14 | 2654.81 | 154.34 | 21.36 | 24.69 | 27.80 | 129.21 | 30.56 | 95 | 0 | 5 |
| BW79 | 8.75 | 19 | 0.01 | 0.19 | 51.70 | 1534.02 | 157.47 | 20.73 | 17.35 | 23.66 | 140.20 | 32.46 | 95 | 0 | 5 |
| BW80 | 6.18 | 10.3333 | 0.03 | 0.31 | 40.61 | 270.22 | 68.78 | 11.46 | 7.92 | 10.35 | 242.65 | 28.19 | 94 | 0 | 6 |
| BW81 | 5.67 | 10.5 | 0.02 | 0.21 | 32.43 | 168.44 | 69.42 | 10.98 | 4.50 | 7.64 | 267.69 | 34.23 | 93 | 0 | 7 |
| BW82 | 5.93 | 10.3333 | 0.03 | 0.31 | 41.24 | 189.97 | 52.33 | 10.89 | 4.55 | 7.92 | 236.04 | 41.00 | 94 | 0 | 6 |
| BW83 | 5.58 | 10.7273 | 0.11 | 1.18 | 46.24 | 423.41 | 72.72 | 13.85 | 14.85 | 37.61 | 194.19 | 39.57 | 92 | 0 | 8 |
| BW84 | 6.13 | 8.25 | 0.04 | 0.33 | 118.39 | 714.51 | 248.83 | 19.26 | 9.41 | 66.50 | 376.52 | 58.57 | 78 | 2 | 20 |
| BW85 | 5.94 | 9 | 0.06 | 0.54 | 200.12 | 498.46 | 256.61 | 20.04 | 12.92 | 61.78 | 411.60 | 68.56 | 80 | 0 | 20 |
| BW86 | 5.61 | 11 | 0.04 | 0.44 | 38.39 | 235.27 | 65.35 | 12.29 | 5.82 | 6.82 | 200.43 | 38.82 | 91 | 0 | 9 |
| BW87 | 7 | 10.5 | 0.08 | 0.84 | 255.09 | 1949.00 | 447.53 | 22.00 | 3.81 | 58.75 | 319.78 | 40.21 | 75 | 5 | 21 |
| BW88 | 7.84 | 10.5556 | 0.09 | 0.95 | 260.28 | 2600.86 | 373.30 | 23.09 | 11.94 | 135.82 | 506.65 | 56.71 | 79 | 3 | 18 |
| BW89 | 5.93 | 11 | 0.05 | 0.55 | 140.94 | 393.20 | 127.43 | 21.21 | 13.24 | 107.66 | 556.99 | 45.25 | 84 | 0 | 16 |
