# Supplementary material for: Microbial diversity in the arid and semi‐arid soils of Botswana
Source: Environ Microbiol Rep. 2024 Nov 13;16(6):e70044. doi: 10.1111/1758-2229.70044 (PMC11558117; doi:10.1111/1758-2229.70044)
Supplement: Supplementary file 3 — TABLE S2: The correlation matrix between environmental variables. Values represent the Spearman's correlation coefficient. TABLE S3: Final selected best subset regression model of the predictors of bacterial species alpha diversity (observed) in the different ecological regions. TABLE S4: Final selected best subset regression model of the predictors of fungal species alpha diversity (observed) in the different ecological regions. TABLE S5: Summary of the redundancy analysis (RDA) results showing significant variables influencing bacterial community structure and diversity in the 89 sampling sites. TABLE S6: Summary of the redundancy analysis (RDA) results showing significant variables influencing fungal community structure and diversity in the 89 sampling sites. TABLE S7: Topological properties of the network of the acidic, alkaline, and neutral networks. TABLE S8: Mean values for the environmental variables for the soils across the different pH categories. [file EMI4-16-e70044-s001.docx]

**Supplementary Tables**

**Table S2**: The correlation matrix between environmental variables. Values represent the Spearman’s correlation coefficient.

|  | **pH** | **CN_R** | **TN** | **TOC** | **K** | **Ca** | **Mg** | **Na** | **P** | **Mn** | **Al** | **Fe** | **NPP** | **MAT** | **MAP** |
| --- | --- | --- | --- | --- | --- | --- | --- | --- | --- | --- | --- | --- | --- | --- | --- |
| **CN_R** | 0.14 |  |  |  |  |  |  |  |  |  |  |  |  |  |  |
| **TN** | 0.04 | **-0.39** |  |  |  |  |  |  |  |  |  |  |  |  |  |
| **TOC** | 0.08 | -0.01 | 0.90 |  |  |  |  |  |  |  |  |  |  |  |  |
| **K** | **0.29** | **-0.28** | **0.73** | **0.64** |  |  |  |  |  |  |  |  |  |  |  |
| **Ca** | 0.75 | -0.05 | **0.57** | **0.55** | **0.69** |  |  |  |  |  |  |  |  |  |  |
| **Mg** | **0.43** | **-0.24** | **0.68** | **0.59** | 0.86 | 0.79 |  |  |  |  |  |  |  |  |  |
| **Na** | **0.57** | -0.04 | **0.55** | **0.56** | **0.73** | 0.86 | 0.79 |  |  |  |  |  |  |  |  |
| **P** | -0.08 | -0.17 | **0.31** | **0.24** | **0.30** | 0.09 | 0.20 | 0.11 |  |  |  |  |  |  |  |
| **Mn** | **0.24** | -0.12 | **0.68** | **0.64** | **0.62** | **0.55** | **0.70** | **0.51** | **0.29** |  |  |  |  |  |  |
| **Al** | **-0.32** | -0.09 | **0.44** | **0.44** | **0.50** | 0.04 | **0.48** | 0.21 | 0.19 | **0.59** |  |  |  |  |  |
| **Fe** | **-0.28** | -0.20 | **0.59** | **0.52** | **0.56** | 0.17 | **0.54** | **0.26** | **0.37** | **0.58** | 0.76 |  |  |  |  |
| **NPP** | 0.01 | 0.20 | **0.29** | **0.42** | 0.05 | 0.18 | 0.06 | 0.18 | -0.08 | 0.21 | -0.01 | 0.17 |  |  |  |
| **MAT** | **0.28** | 0.05 | 0.18 | 0.22 | 0.05 | **0.28** | 0.05 | **0.27** | -0.09 | 0.04 | **-0.29** | -0.10 | **0.46** |  |  |
| **MAP** | **-0.24** | 0.09 | **0.32** | **0.42** | 0.02 | 0.07 | 0.07 | 0.09 | -0.09 | **0.28** | **0.25** | **0.36** | **0.48** | 0.12 |  |
| **A In** | -0.19 | **0.24** | **0.34** | **0.50** | 0.14 | 0.13 | 0.17 | **0.23** | -0.04 | **0.42** | **0.39** | **0.40** | **0.47** | 0.09 | 0.87 |

Bold values represent significant correlation (p<0.05)

**Table S3**: Final selected Best subset regression model of the predictors of bacterial species alpha diversity (Observed) in the different ecological regions.

| Predictor | Estimate | Standard error | Std.Error | Pr(>\|t\|) |
| --- | --- | --- | --- | --- |
| Intercept | 6.85 | 1.15 x 10^-1^ | 59.58 | <2 x 10^-16^*** |
| pH | 2.99 x 10^-2^ | 1.48 x 10^-2^ | 2.02 | 4.68 x 10^-2^* |
| Mg | -1.38 x 10^-4^ | 1.06 x 10^-4^ | -1.31 | 1.9577 x 10 |
| Na | 3.47 x 10^-3^ | 1.26 x 10^-3^ | 2.75 | 7.56 x 10^-3^** |
| NPP | 4.69 x 10^-5^ | 1.91 x 10^-5^ | 2.45 | 1.666 x 10^-2^* |
|  |  |  |  |  |
| Adjusted R squared | 0.25 |  |  |  |
| BIC | -4.54 |  |  |  |
| Mallow’s cp | 1.16 |  |  |  |
| cv.errors | 171.55 |  |  |  |

The best models were selected based on: (i) high adjusted R^2^ values, (ii) Mallow Cp value that is close to the number of predictor variables, and (iii) goodness of fit of the residual plot. Significant differences are based on the ANOVA and are represented by asterisks as follows: ***P<0.001 **P<0.01 *P< 0.05.

**Table S4**: Final selected Best subset regression model of the predictors of fungal species alpha diversity (Observed) in the different ecological regions.

| Predictor | Estimate | Standard error | Std.Error | Pr(>\|t\|) |
| --- | --- | --- | --- | --- |
| Intercept | 5.62 | 1.02 x 10^-1^ | 55.08 | < 2 x 10^-16^*** |
| pH | 1.67 x 10^-1^ | 6.30 x 10^-2^ | 2.65 | 9.92 x 10^-3^** |
| Mg | 2.10 x 10^-3^ | 5.70 x 10^-4^ | 3.69 | 4.33 x 10^-4^*** |
| Na | -1.60 x 10^-3^ | 9.70 x 10^-4^ | -1.65 | 1.028 x 10^-1^ |
| NPP | 1.35 x10^-4^ | 3.31 x 10^-5^ | 4.07 | 1.19 x 10^-4^*** |
|  |  |  |  |  |
| **Adjusted R squared** | 0.40 |  |  |  |
| **BIC** | -22.26 |  |  |  |
| **Mallow’s cp** | 3.28 |  |  |  |
| **cv.errors** | 90.95 |  |  |  |

The best models were selected based on: (i) high adjusted R^2^ values, (ii) Mallow Cp value that is close to the number of predictor variables, and (iii) goodness of fit of the residual plot. Significant differences are based on the ANOVA and are represented by asterisks as follows: ***P<0.001 **P<0.01 *P< 0.05.

**Table S5**: Summary of the Redundancy analysis (RDA) results showing significant variables influencing bacterial community structure and diversity in the 89 sampling sites.

|  | Variance | F | Pr(>F) |
| --- | --- | --- | --- |
| pH | 0.076 | 11.353 | 0.001 *** |
| Ca | 0.014 | 2.174 | 0.003 ** |
| Na | 0.015 | 2.278 | 0.001 *** |
| MAP | 0.013 | 1.924 | 0.009 ** |
| Al | 0.019 | 2.838 | 0.001 *** |
| CN_R | 0.011 | 1.655 | 0.017 * |
| C | 0.018 | 2.678 | 0.001 *** |
| P | 0.011 | 1.624 | 0.024 * |
| Mg | 0.011 | 1.627 | 0.014 * |
|  |  |  |  |
|  | **Inertia** | **Proportion** |  |
| Total | 0.648 | 1.000 |  |
| Constrained | 0.188 | 0.290 |  |
| Unconstrained | 0.460 | 0.710 |  |

Significant differences are based on the ANOVA and are represented by asterisks as follows: ***P<0.001 **P<0.01 *P< 0.05.

**Table S6**: Summary of the Redundancy analysis (RDA) results showing significant variables influencing fungal community structure and diversity in the 89 sampling sites.

|  | Variance | F | Pr(>F) |
| --- | --- | --- | --- |
| MAP | 0.028 | 3.396 | 0.001 *** |
| pH | 0.026 | 3.184 | 0.001 *** |
| Mn | 0.018 | 2.152 | 0.001 *** |
| MAT | 0.015 | 1.845 | 0.001 *** |
| Mg | 0.012 | 1.408 | 0.008 ** |
| Na | 0.014 | 1.653 | 0.001 *** |
| P | 0.011 | 1.353 | 0.012 * |
| CN_R | 0.012 | 1.507 | 0.005 ** |
| Ca | 0.013 | 1.626 | 0.001 *** |
| C | 0.011 | 1.343 | 0.014 * |
|  |  |  |  |
|  | **Inertia** | **Proportion** |  |
| Total | 0.790 | 1.000 |  |
| Constrained | 0.161 | 0.204 |  |
| Unconstrained | 0.629 | 0.796 |  |

Significant differences are based on the ANOVA and are represented by asterisks as follows: ***P<0.001 **P<0.01 *P< 0.05.

**Table S7:** Topological properties of the network of the acidic, alkaline, and neutral networks

| Sample | Acidic network | Alkaline network | Neutral network |
| --- | --- | --- | --- |
| Total nodes | 1013 | 1006 | 895 |
| Total links | 2743 | 2627 | 2210 |
| Positive interactions | 99% | 99% | 97% |
| Negative interaction | 1.0% | 1.0% | 3% |
| Average network degree | 5.42 | 5.22 | 4.94 |
| Diameter | 16 | 17 | 18 |
| Average path length | 6.266 | 6.249 | 6.147 |
| Modularity index | 0.749 | 0.723 | 0.70 |
| Average clustering coefficient | 0.31 | 0.30 | 0.32 |
| No of communities | 51 | 59 | 83 |

Table S8: Mean values for the environmental variables for the soils across the different pH

categories.

|  | **Acidic** | **Alkaline** | **Neutral** | **p-value** |
| --- | --- | --- | --- | --- |
| pH | 6.02±0.41a | 8.42±0.62b | 6.99±0.20c | 3.38 x 10^-4^*** |
| CN-R | 13.2±5a | 14±4.75a | 12±2.43a | 3.2 x 10^-1^ |
|  |  |  |  |  |
| **Concentration (mg/kg) of:** | |  |  |  |
| Potassium (K) | 109±109a | 225±335b | 129±130ab | 1.44 x 10^-2^* |
| Calcium (Ca) | 585±1029a | 3606±2375b | 1134±1060c | 6.26 x 10^-11^*** |
| Magnesium (Mg) | 130±171a | 340±515b | 237±280b | 2.78 x 10^-4^*** |
| Sodium (Na) | 21.4±24.6a | 677±2866b | 22.2±17a | 1.16 x 10^-6^*** |
| Phosphorus (P) | 11.3±8.55a | 12.5±11.3a | 11.6±11.2a | 0.86 x 10^-1^ |
| Manganese (Mn) | 31.1±28.7a | 48.8±35.9b | 47.9±45.3ab | 4.83 x 10^-2^* |
| Aluminium (Al) | 292±170a | 205±202b | 268±134ab | 1.99E x 10^-2^* |
| Iron (Fe) | 65.3±98a | 32.9±15.9b | 50±48ab | 5.16 x 10^-2^* |
|  |  |  |  |  |
| **% of:** |  |  |  |  |
| Total organic carbon (TOC) | 0.61±0.55a | 0.63±0.28a | 0.72±0.58a | 4.14 x 10^-1^ |
| Total nitrogen (TN) | 0.060±0.09a | 0.051±0.03a | 0.062±0.06a | 4.26 x 10^-1^ |
| Sand | 84.6±14.7a | 80.7±18.8a | 83.4±13.7a | 5.89 x 10^-1^ |
| Silt | 2.43±9.41a | 1.67±3.03a | 1.72±3.24a | 4.09 x 10^-1^ |
| Clay | 13±8.39a | 17.6±16.7a | 14.9±13a | 6.58 x 10^-1^ |
|  |  |  |  |  |
| NPP | 2833±640a | 2911±890a | 2745±535a | 5.55 x 10^-1^ |
| MAT | 203±9.8a | 205±35.7b | 201±35.6ab | 1.39 x 10^-2^* |
| MAP | 434±60.5a | 401±98.4ab | 390±50b | 1.87 x 10^-2^* |
| Aridity index | 0.174±0.03a | 0.158±0.04a | 0.153±0.03a | 9.38 x 10^-2^* |

Values are means (± standard deviation). Significant differences are based on the Wilcoxon rank-sum test and are represented by asterisks as follows: ***P<0.001 **P<0.01 *P< 0.05. Pairwise differences were generated using Dunn’s Test.
